# Comparative Outcomes of Resurfacing vs Total Hip Arthroplasty: A Systematic Review and Meta‐Analysis

**DOI:** 10.1155/aort/4661645

**Published:** 2026-07-07

**Authors:** Christoforos Demosthenous, Christos Pittalis, Gregoris A. Orphanides, Konstantinos Antoniou, Efthimios Demetriou, Petros Kyriakou, Vasilis Stylianides, Foteini Moniati

**Affiliations:** ^1^ Barts and the London School of Medicine and Dentistry, Queen Mary University of London, London, UK, qmul.ac.uk; ^2^ School of Medicine, University of Dundee, Dundee, UK, dundee.ac.uk; ^3^ Department of Medicine, University Hospitals Birmingham NHS Trust, Birmingham, UK

## Abstract

**Background:**

Total hip arthroplasty (THA) and resurfacing hip arthroplasty (RHA) are widely used for treating hip joint disorders. While THA is the gold standard for hip replacement, RHA is increasingly used in younger, active patients due to bone preservation. This systematic review and meta‐analysis compare functional outcomes, metal ions, complications, revision rates, survivorship and satisfaction between THA and RHA.

**Methods:**

A systematic search of EMBASE, Cochrane Library and PubMed was conducted (inception–August 2024) to identify primary research articles for THA and RHA. Risk of bias was assessed using the Newcastle–Ottawa Quality Assessment Scale. A meta‐analysis using MATLAB applied a random‐effects model to calculate pooled effect sizes for functional scores, revision rates, complications and metal ions.

**Results:**

Initial search collated 2265 studies, of which 11 met inclusion criteria (2013–2021). Most frequently reported outcomes included complications, revision rates and UCLA. RHA showed better UCLA, HHS and chromium levels, while THA had better WOMAC and cobalt levels, although differences were nonsignificant. Total complications were higher with THA, though nonsignificant. The most common complications were aseptic loosening and dislocation for RHA and THA, respectively. Notably, when the dislocation rate was analysed in isolation, a statistically significant higher dislocation rate was demonstrated with THA. Revision rates were higher for THA, and survivorship was 85.5% compared to 89.5% (RHA), however, with no statistical difference. Statistically significant higher preference for RHA (66.1%) was reported in one study. Satisfaction was greater for RHA (87.8% vs 83.9%) and more patients perceived their hip as natural. One study reported significantly higher quality of life with THA.

**Conclusion:**

Patient‐reported outcomes, metal ions, complications, revision rates and survivorship were broadly similar between RHA and THA. However, dislocations were more frequent with THA, a key consideration when treating athletic patients. Greater satisfaction and preference with RHA suggest that it may be preferable when outcomes are otherwise comparable.

Christoforos Demosthenous and Christos Pittalis are joined‐first authors.

## 1. Introduction

Hip arthroplasty is widely used to relieve pain and restore function in patients with hip joint damage, commonly from fractures, rheumatoid arthritis or osteoarthritis. Total hip arthroplasty (THA) remains the gold‐standard treatment for end‐stage hip arthritis, offering substantial pain relief, functional improvement, enhanced quality of life [1] and implant survivorship of 90–95% at 15 years [2]. Outcomes and patient satisfaction for THA have been significantly enhanced through advancements in surgical techniques, implant architecture and rehabilitation regimens [3]. Despite these advancements, younger patients face a higher lifetime risk of revision due to implant wear [4], and although THA enables patients to resume low‐impact activities, high‐impact sports are generally contraindicated due to joint mobility limitations [5].

Resurfacing hip arthroplasty (RHA) has been developed for younger, active patients, offering bone preservation. The mechanism behind hip resurfacing involves capping the femoral head with a metal component and resurfacing the acetabulum with a metal cup. It enables patients to return to high levels of physical activity and sports [6], evident through the study of Fouilleron et al., which reported that 92% of patients went back to running [7]. Similarly, 98% of patients returned to a mean of 4.6 athletic disciplines, with the majority resuming sports within the first 3 months following surgery according to Naal et al. [8]. Newer designs, such as the Birmingham hip resurfacing (BHR), have reported around 90% survivorship at 10 years [9]. Despite benefits like greater range of motion and lower dislocation risk compared to THA, concerns remain regarding metal ion release. As outcomes tend to be better in younger males with larger bone morphology, patient selection remains critical, since implants are usually designed for male anatomy [10].

Prosthetic bearing surfaces utilised in hip arthroplasties include metal‐on‐metal (MoM), metal‐on‐polyethylene (MoP), ceramic‐on‐ceramic (CoC) and ceramic‐on‐polyethylene (CoP). While MoM bearings have largely been abandoned in THA due to catastrophic adverse events such as metal ion toxicity and pseudotumour formation, it remains integral in RHA design [11, 12]. Although the optimal femoral head material remains debated, metal‐on‐HXLPE is the most clinically established option. In younger patients, bearing surface choice remains contentious. Although polyethene is often favoured for its ease of use, concerns about wear and aseptic loosening persist [13].

There are limited data comparing the outcomes and complications between THA and RHA. Within this context, this systematic review and meta‐analysis aim to critically evaluate functional patient‐reported scores, complications, revision rates, survivorship, metal ion levels and satisfaction between THA and RHA, while determining whether clinical results reflect the proposed advantages of RHA.

## 2. Methods

### 2.1. Literature Search Strategy

The Preferred Reporting Items for Systematic Reviews and Meta‐Analyses (PRISMA) checklist and flow diagram were followed [14]. A completed PRISMA checklist is available in Supporting Appendix S4. Prospective registration in PROSPERO was not possible due to the retrospective nature of the study; therefore, the review protocol was retrospectively registered with the Open Science Framework (OSF; https://doi.org/10.17605/OSF.IO/2UJ3G) as an alternative platform. A comprehensive literature search was performed including searches in EMBASE, PubMed and Cochrane covering the period from their establishment up to and including August 2024. To make sure that we included all the relevant studies, the appropriate MeSH search terms were used to device a universal search question: (‘total hip arthroplasty′/exp OR ‘total hip arthroplasty’ OR tha OR thr) AND (‘hip resurfacing arthroplasty′/exp OR ‘hip resurfacing arthroplasty’ OR ‘hip resurfacing’ OR hra OR rha) AND (compar^∗^ OR ‘and’ OR versus OR vs OR outcome). Full Boolean queries used in each database are provided in Supporting Appendix S1.

### 2.2. Inclusion and Exclusion Criteria

Defined inclusion criteria were employed for this study. These consisted of primary research studies, such as randomised controlled trials and comparative observational studies, pertaining to and comparing the outcomes of THA versus RHA in human patients. No minimum mean or median follow‐up period was set. No specific outcome measures were predefined for inclusion prior to the literature search. This methodological choice was deliberate and reflects the exploratory and heterogenous nature of the existing THA versus RHA literature, in which there is no established consensus on a single primary or core outcome set. Predetermining a narrow set of outcomes risked excluding clinically important endpoints that may be captured differently across studies. Instead, an empirical, frequency‐based approach was adopted: All eligible comparative studies were included, and the outcomes most consistently reported across the included studies were subsequently identified and analysed, specifically UCLA, Harris hip score (HHS), WOMAC, complications, metal ion levels, revision rates, survivorship and patient satisfaction. Search parameters were limited to studies published in the English language and accessible as full‐text articles in peer‐reviewed journals.

Exclusion criteria comprised secondary research studies such as case series, case reports, review articles, editorials and conference abstracts. Additionally, studies not directly comparing THA and RHA, lacking full‐text access, or not published in the English language were also excluded.

### 2.3. Data Extraction

Study selection was initially conducted independently by two reviewers to minimise selection bias. A consensus was first established regarding the research question, and MeSH terms were used during the screening process. Titles and relevant abstracts were screened by the same two reviewers, with full‐text reviews performed when additional evaluation was necessary. In instances of disagreement over study inclusion, a third independent reviewer was consulted, and a voting system was employed to decide whether the study should be included or excluded from the analysis.

Data from the eligible studies were diligently extracted and recorded into an electronic screening form by the same two reviewers to ensure consistency and reliability. The data collected from each article included authors’ names, publication year, study type, sample size, demographic characteristics, number of patients who received RHA and THA procedures, complications, functional scores (WOMAC, UCLA and HHS), metal ion levels, survivorship, preference and satisfaction. Any disagreements during data extraction were resolved by consensus or consultation with the third reviewer.

### 2.4. Methodological Quality Assessment

The quality of the studies included in this review was independently evaluated by two authors using the Newcastle–Ottawa Quality Assessment Scale (NOS) [15]. This tool is specifically designed to assess studies based on three domains: selection, comparability and outcomes. For the selection (four items) and outcome (three items) domains, each study could receive a maximum of one star (^∗^) per item. In the comparability domain, studies could receive up to two stars per item. A higher total star rating indicated higher study quality. Any unresolved disagreements between reviewers were addressed and resolved through consensus.

### 2.5. Statistical Analysis

A meta‐analysis was conducted to assess the difference between the outcomes of RHA and THA using MATLAB R Version 2023b [16]. For the functional outcomes (UCLA, WOMAC and HHS) assessed in the statistical analyses and the metal ion levels, the effect size was computed using the mean difference (MD). For the revision rates and complications (total, fractures, dislocations and adverse reaction to metal debris [ARMD]), the effect sizes were computed using odds ratios (ORs). When not directly reported, effect sizes were calculated from raw data by using custom‐developed code in MATLAB. The inverse‐variance weighting method was employed to calculate study weights, and heterogeneity was assessed using the tau^2^, chi^2^ and *I* ^2^ statistics. A random‐effects model was utilised, and the pooled effect sizes and 95% CIs were computed. Finally, the data from the studies were combined to obtain the overall effect size and its 95% CI and calculate the *Z* statistic. The statistical significance was set as *p* < 0.05. Graphical outputs (forest plots) were generated using MATLAB’s plotting tools. Subsequently, subgroup and sensitivity analyses were planned to explore potential sources of variability across the included studies, specifically based on bearing surface (MoM THA versus non‐MoM THA), THA femoral head size, age, sex, study type (randomised controlled trials versus observational) and mean follow‐up duration. Where subgroup or sensitivity analyses could not be performed, this is explicitly stated and justified.

### 2.6. Grading of Recommendations Assessment, Development and Evaluation (GRADE) Assessment of Certainty of Evidence

The certainty of evidence for each outcome was assessed using the GRADE approach, following Cochrane guidance [17]. Certainty was assessed across the following five domains: risk of bias, inconsistency, indirectness, imprecision and publication bias. For each outcome, the initial certainty rating was determined according to the study design contributing the greatest weight of evidence. Outcomes for which the greatest weight of evidence came from randomised controlled trials were initially rated as high certainty, while outcomes predominantly informed by nonrandomised evidence would have started at low certainty. Certainty was then downgraded where appropriate based on outcome‐specific concerns. For dichotomous outcomes, anticipated absolute effects were calculated using the assumed risk in the THA group and the pooled OR. For continuous outcomes, comparator values were presented as the mean postoperative value in the THA group and the pooled MD with RHA.

## 3. Results

### 3.1. Description of Studies

**FIGURE 1:**
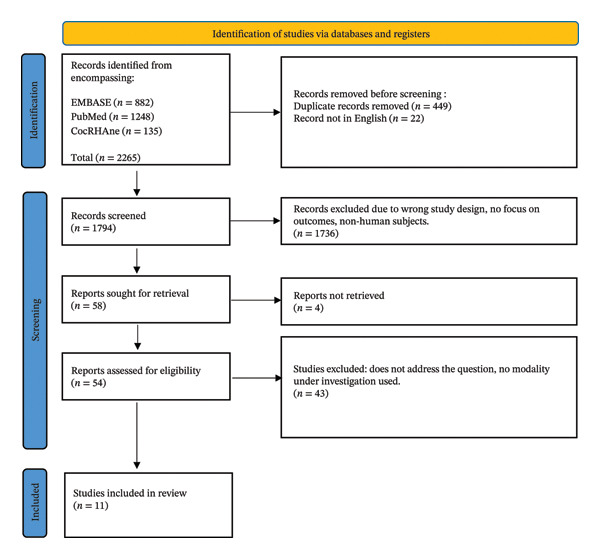
PRISMA flow diagram of the selected studies.

The initial database search yielded a total of 2265 studies. Following the removal of duplicates using Microsoft Excel’s ‘Remove duplicates’ function and manual verification, otherwise ineligible records were excluded, leaving 1794 studies. Following abstract screening, 54 full‐text reports were assessed for eligibility, resulting in 11 studies published between 2013 and 2021 to be included in the final review. The PRISMA flowchart outlining the reasons for exclusion is presented in Figure 1.

### 3.2. Baseline Characteristics

This systematic review included a total of 956 patients (505 underwent RHA and 513 underwent THA). A summary of the baseline characteristics of the selected studies is provided in Table 1, while Table 2 presents a summary of the bearing surface types used in the selected studies. Patient age was reported in all studies, and the mean ranged from 32.2 to 59.4 and 27.9 to 63 for RHA and THA, respectively. Gender was reported in all studies as well, resulting in a male‐to‐female ratio of 630/316 (343/156 in the RHA group and 325/184 in the THA group). The majority of the studies were single‐centred (8/11), while three of them were conducted across multiple centres. The follow‐up period ranged from 6 weeks to 22 years (means of 2–15 years), and the mean follow‐up time across all studies was 7.4 years. The most commonly reported outcomes were complications (all studies), revision rates (all studies), UCLA (8 studies), HHS (7 studies), WOMAC (5 studies), survivorship (5 studies), metal ion levels (4 studies) and OHS (4 studies). Other reported outcomes included SF36, VAS, EQ‐5D, SF12 and FJS scores. Nine studies reported on the surgical approach utilised: 5 posterolateral and 4 posteriors. Indications to surgery were documented in 10 out of 11 studies with the most common being osteoarthritis (10 studies), avascular necrosis (5 studies) and hip dysplasia (3 studies).

**TABLE 1:** Summary of the baseline characteristics of the selected studies.

Author/year	Study type	Treatment group	No. of patients	Total no. of patients	Mean age (years)	Male: female ratio	BMI	Mean follow‐up (years)
Calkins et al. 2021 [18]	Retrospective cohort study	RHA	62	62	51.5	38/24	28.5	11.0
		THA	62		56.6	38/24	29.6	6.0

LeBrun et al. 2021 [19]	Retrospective cohort study	RHA	65	91	32.2	57/8	26.2	2.0
		THA	26		27.9	0/26	23.3	

Hersnaes et al. 2021 [20]	Randomised controlled trial	RHA	36	75	59.4	26/10	27.5	5.0
		THA	39		61.9	26/13	28.4	

Konan et al. 2021 [21]	Prospective randomised controlled trial	RHA	48	104	51.5	43/5	28.3	9.0
		THA	56		52.0	50/6	28.2	

Kostretzis et al. 2021 [22]	Randomised controlled trial	RHA	24	48	50.0	14/10	28.0	14.0
		THA	24		50.0	15/9	28.0	

Vendittoli et al. 2020 [23]	Randomised controlled trial	RHA	104	203	48.9	66/38	26.6	15.0
		THA	99		50.7	67/32	30.0	

Costa et al. 2018 [24]	Randomised controlled trial	RHA	60	122	56.5	36/24	28.4	5.0
		THA	62		56.7	35/27	28.9	

Tao et al. 2018 [25]	Prospective comparative study	RHA	28	68	43.0	19/9	21.5	7.4
		THA	40		47.0	28/12	21.8	

Bisseling et al. 2015 [26]	Randomised controlled trial	RHA	38	71	57.5	21/17	26.1	4.8
		THA	33		59.2	21/12	28.0	

Karampinas et al. 2014 [27]	Randomised controlled trial	RHA	20	41	54.9	11/3	31.0	2.0
		THA	21		57.1	12/5	31.6	

Penny et al. 2013 [28]	Randomised clinical trial	RHA	20	71	57.0	12/8	28.0	2.0
		THA	51		58.0	33/18	27.3	

**TABLE 2:** Summary of bearing surface types of the selected studies.

Author/year	Treatment type	Bearing surface
Calkins et al. 2021 [18]	RHA	MoM
	THA	MoM/CoC/oxinium (28–58 mm)

LeBrun et al. 2021 [19]	RHA	MoM
	THA	CoP/CoC/MoM (32 mm)

Hersnaes et al. 2021 [20]	RHA	MoM
	THA	MoM large‐head

Konan et al. 2021 [21]	RHA	MoM
	THA	MoM large‐head

Kostretzis et al. 2021 [22]	RHA	MoM
	THA	MoM large‐head

Vendittoli et al. 2020 [23]	RHA	MoM
	THA	MoM 28 mm

Costa et al. 2018 [24]	RHA	MoM
	THA	N/A

Tao et al. 2018 [25]	RHA	MoM
	THA	MoM large‐head

Bisseling et al. 2015 [26]	RHA	MoM
	THA	MoM 28 mm

Karampinas et al. 2014 [27]	RHA	MoM
	THA	MoM large‐head

Penny et al. 2013 [28]	RHA	MoM
	THA	MoM large‐head/MoP, CoP 28 mm

### 3.3. Methodological Quality Assessment

Table 3 outlines the findings of the methodological quality of bias assessment.

**TABLE 3:** Quality of bias assessment scoring (NOS).

Study ID	Selection				Comparability	Outcome			Total (9^∗^)
	(a)	(b)	(c)	(d)	(e)	(f)	(g)	(h)	
Calcins et al. 2021 [18]	1	0	1	1	1	1	1	1	7
LeBrun et al. 2021 [19]	1	0	1	1	1	1	1	1	7
Hersnaes et al. 2021 [20]	1	0	1	1	1	1	1	1	7
Konan et al. 2021 [21]	1	0	1	1	1	1	1	1	7
Kostretzis et al. 2021 [22]	1	0	1	1	1	1	1	1	7
Vendittoli et al. 2020 [23]	1	0	1	1	1	1	1	1	7
Costa et al. 2018 [24]	1	0	1	1	1	1	1	1	7
Tao et al. 2018 [25]	1	0	1	1	1	1	1	1	7
Bisseling et al. 2015 [26]	1	0	1	1	1	1	1	1	7
Karampinas et al. 2014 [27]	1	0	1	1	1	1	1	1	7
Penny et al. 2013 [28]	1	0	1	1	1	1	1	1	7

*Note:* (a) Representativeness of the exposed cohort (^∗^), (b) selection of the nonexposed cohort (^∗^), (c) ascertainment of exposure (^∗^), (d) demonstration that the outcome of interest was not present at the start of the study (^∗^), (e) comparability of cohorts (^∗∗^), (f) assessment of outcome (^∗^), (g) follow‐up was long enough for outcomes to occur (^∗^) and (h) adequacy of follow‐up (^∗^).

### 3.4. Outcomes and Data Availability

Table 4 summarises the outcomes included in the analysis and the corresponding data availability across studies.

**TABLE 4:** Summary of the outcomes included and data availability.

Outcome	Studies (*n*)	Studies in main meta‐analysis (*n*)	Dataset	Reason for reduced dataset	Sensitivity analysis performed (Y/N)
UCLA	8	8	Near‐complete	Not reported in remaining studies	Yes
HHS	7	7	Subset	Not reported in remaining studies	Yes
WOMAC	5	5	Subset	Not reported in remaining studies	Yes
Metal ion levels (cobalt)	4	3	Subset	Not reported in remaining studies	Yes
Metal ion levels (chromium)	3	3	Subset	Not reported in remaining studies	Yes
Total complications	10	9	Near‐complete	Not reported in remaining studies	Yes
Fractures	3	3	Subset	Not reported in remaining studies	Yes
Dislocations	4	4	Subset	Not reported in remaining studies	Yes
ARMD	3	3	Subset	Not reported in remaining studies	Yes
Aseptic loosening	5	5	Subset	Not reported in remaining studies	Yes
Revision rates	10	10	Near‐complete	Not reported in remaining studies	Yes
Survivorship	4	N/A	Subset	Not reported in remaining studies	N/A
Satisfaction or quality of life	6	N/A	Subset	Not reported in remaining studies	N/A

### 3.5. Functional Patient‐Reported Outcomes

#### 3.5.1. UCLA

The mean preoperative UCLA score was reported in 4 studies [19, 26–28], ranging from 4.07 to 5.8 in the RHA group and 3.2 to 6.3 in the THA group, as shown in Table 5. The mean preoperative UCLA score across the 4 studies was 5 and 4.2 for the RHA and THA groups, respectively. The mean postoperative UCLA score was reported in 8 studies for RHA [19, 21–23, 25–28] and 8 for THA [19, 21–23, 25–28], ranging from 6.3 to 8.5 in the RHA group and 5.9 to 8.7 in the THA group. A mean postoperative UCLA score of 7.4 was obtained for the RHA group and 6.9 for THA across the reporting studies. In total, 4 studies reported higher postoperative UCLA scores for the RHA group [19, 21, 22, 27] compared to 2 studies that reported higher scores for the THA group [23, 25], while 2 studies reported the same scores among the groups [26, 28]. However, only 2 studies showed statistical significance, both favouring the RHA group [23, 26]. One study reported a statistically significant decline in the UCLA score from 1 year to the last follow‐up and another from 5 years to last follow‐up [22, 23].

**TABLE 5:** Summary of all functional patient‐reported outcomes.

Author/year	Treatment type	WOMAC preop	WOMAC postop	UCLA preop	UCLA postop	HHS (Harris hip score) preop	HHS postop
Calkins et al. 2021 [18]	RHA	N/A	N/A	N/A	N/A	54.1 (9.3)	90.7 (8.7)
	THA	N/A	N/A	N/A	N/A	55.8 (10.1)	86.9 (12.1)

LeBrun et al. 2021 [19]	RHA	N/A	N/A	5.2 (2.4)	8.4 (1.6)	58.9 (14.3)	94.4 (8.2)
	THA	N/A	N/A	3.2 (1.1)	6.7 (2.3)	50.2 (8.6)	86.7 (13.5)

Hersnaes et al. 2021 [20]	RHA	N/A	N/A	N/A	N/A	N/A	100 (93–100)
	THA	N/A	N/A	N/A	N/A	N/A	100 (98–100)

Konan et al. 2021 [21]	RHA	51.1	88.6 (13.4)	N/A	6.5 (1.9)	N/A	N/A
	THA	52.6	88 (15.7)	N/A	5.9 (1.7)	N/A	N/A

Kostretzis et al. 2021 [22]	RHA	N/A	85.0 (16.0)	N/A	7.2 (1.8)	N/A	N/A
	THA	N/A	94.0 (7.8)	N/A	6.7 (1.8)	N/A	N/A

Vendittoli et al. 2020 [23]	RHA	52.7	89.3 (6.2)	N/A	6.3 (2.0)	N/A	N/A
	THA	55.0	91.2 (11.8)	N/A	6.4 (4.6)	N/A	N/A

Costa et al. 2018 [24]	RHA	N/A	N/A	N/A	N/A	N/A	N/A
	THA	N/A	N/A	N/A	N/A	N/A	N/A

Tao et al. 2018 [25]	RHA	N/A	N/A	N/A	8.5 (1.0)	50.5 (41.0–62.0)	90.4 (2.4)
	THA	N/A	N/A	N/A	8.7 (1.3)	49.2 (42.0–59.0)	90.8 (5.1)

Bisseling et al. 2015 [26]	RHA	N/A	N/A	5.0 (4.0–7.0)	7.0 (7.0–8.0)	57 (54.0–60.0)	98.0 (96–100)
	THA	N/A	N/A	4.0 (4–4)	7.0 (6–8)	53 (44.0–59.0)	96.0 (93–100)

Karampinas et al. 2014 [27]	RHA	72.4 (10.2)	94.6 (3.0)	4.1 (1.5)	8.1 (1.1)	60.3 (9.9)	95.7 (2.0)
	THA	65.6 (10.9)	93.4 (34.8)	3.5 (1.2)	6.8 (1.1)	56.5 (11.9)	93.8 (3.6)

Penny et al. 2013 [28]	RHA	50.0 (21.0)	92.0 (13.0)	5.8 (2.2)	7.3 (1.8)	63.0 (10.0)	93.0 (10.0)
	THA	55.0 (16.0)	91.7 (15.3)	6.3 (1.8)	7.3 (1.9)	56.0 (9.0)	92.4 (11.3)
	LHTHA	49.0 (20.0)		5.8 (1.6)		59.0 (8.0)	

*Note:* Number in brackets represents the standard deviation or the range.

**FIGURE 2:**
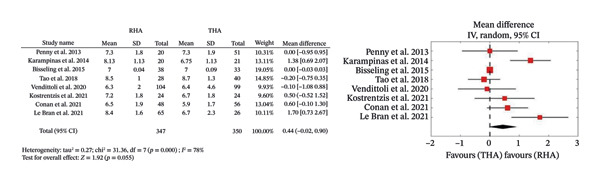
Forest plot of postoperative UCLA scores. IV = inverse variance.

Eight studies were included in the meta‐analysis of postoperative UCLA scores, which is portrayed in Figure 2. The pooled MD between UCLA scores in RHA and THA was 0.44 (95% CI: −0.02, 0.90; *p* = 0.055). This value favours the RHA group but without statistical significance. Heterogeneity across studies was very high with an *I* ^2^ value of 78%.

#### 3.5.2. HHS

The mean preoperative HHS was used in 6 studies [18, 19, 25–28], with values ranging between 50.5 and 63 for RHA and 49.2 to 59 for THA, as shown in Table 5. The mean preoperative HHS was 57.3 in the RHA group and 53.6 in the THA group. The postoperative HHS was utilised in 7 studies for RHA [18–20, 25–28] and 7 studies for THA [18–20, 25–28], ranging from 90.4 to 100 and 86.7 to 100, respectively. The mean postoperative HHS for the RHA group was 94.6, and that for the THA group was 92.4. In total, 5 studies reported higher postoperative HHSs for the RHA group [18, 19, 26–28] compared to only 1 study for the THA [25] and 1 that reported the same scores [20]. Only 2 studies observed statistically significant HHSs at 6 months versus last follow‐up, both favouring the RHA group [18, 26].

**FIGURE 3:**
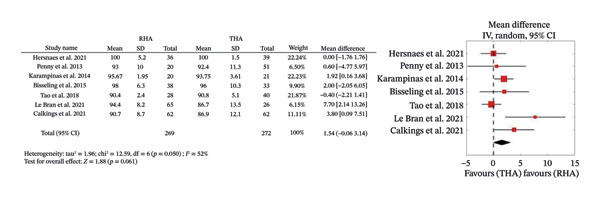
Forest plot of postoperative HHSs. IV = inverse variance.

Seven studies were included in the meta‐analysis of postoperative HHSs, as shown in Figure 3. The pooled MD was 1.54 (95% CI: −0.06, 3.14; *p* = 0.061). This value favours the RHA group, but without statistical significance. Heterogeneity across studies was high with an *I* ^2^ value of 52%.

The mean preoperative WOMAC score was reported in 4 studies [21, 23, 27, 28], ranging from 50 to 72.4 in the RHA group and 49 to 65.6 in the THA group, as shown in Table 5. The mean preoperative WOMAC score across the 4 studies was 56.5 for both the RHA and THA groups. The postoperative WOMAC score was obtained in 5 studies [21–23, 27, 28], with values ranging from 85 to 94.6 in the RHA group and 88 to 95 in the THA group. The mean postoperative WOMAC score was calculated as 89.9 and 91.7 for RHA and THA groups, respectively. However, in total, 3 studies reported higher WOMAC scores for the RHA group [21, 27, 28] compared to 2 studies that reported higher WOMAC scores for the THA group [22, 23]. Statistical significance was observed only in 1 study, favouring the THA group [22]. Another study reported statistically significant deterioration between 5 years and the last follow‐up [23].

**FIGURE 4:**
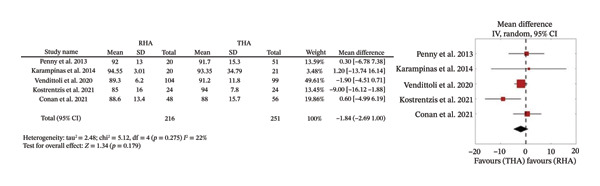
Forest plot of postoperative WOMAC scores. IV = inverse variance.

Fifth studies were incorporated in the meta‐analysis of postoperative WOMAC scores, as evident in Figure 4. The pooled MD was 1.84 (95% CI: −2.69, 1.00; *p* = 0.179). This value favours the THA group, but there is no statistical significance. Heterogeneity across the 5 studies was low with an *I* ^2^ value of 22%.

### 3.6. Metal Ion Levels

The preoperative metal ion levels, cobalt and chromium, were measured in only 2 studies for the RHA group [19, 26] with a mean of 0.85 μg/L and 1.1 μg/L, respectively, and 1 study for the THA group [22], with a mean of 0.1 μg/L for both ions. The postoperative levels were reported in 4 and 3 studies for RHA [19, 20, 22, 26] and THA [20, 22, 26] groups, respectively. The difference in the number of datasets reflects that not all included studies reported outcomes for both intervention groups, with some studies providing data for RHA only. The mean postoperative level of cobalt was 1.35 μg/L for the RHA group and 2.12 μg/L for the THA group. The mean postoperative level of chromium was 1.35 μg/L for the RHA group and 1.12 μg/L for the THA group. A summary of all metal ion levels is provided in Table 6. In the THA group, Refs. [20, 22] used large‐diameter head MoM bearings, whereas Ref. [26] used a 28‐mm MoM bearing.

**TABLE 6:** Summary of all metal ion levels.

Author/year	Treatment type	Cobalt preop	Cobalt postop	Chromium preop	Chromium postop
Calkins et al. 2021 [18]	RHA	N/A	N/A	N/A	N/A
	THA	N/A	N/A	N/A	N/A

LeBrun et al. 2021 [19]	RHA	1.6 (1.2–2.7)	1.5 (1.2–2.9)	2.1 (1.5–3.7)	1.9 (1.4–3.6)
	THA	N/A	N/A	N/A	N/A

Hersnaes et al. 2021 [20]	RHA	N/A	0.9 (0.6–1.5)	N/A	1.2 (0.9–3.0)
	THA	N/A	1.7 (0.9–2.3)	N/A	1.4 (1.0–3.1)

Konan et al. 2021 [21]	RHA	N/A	N/A	N/A	N/A
	THA	N/A	N/A	N/A	N/A

Kostretzis et al. 2021 [22]	RHA	N/A	1.7 (2)	N/A	1.4 (1.1)
	THA	N/A	3.8 (3.2)	N/A	1.9 (1.0)

Vendittoli et al. 2020 [23]	RHA	N/A	N/A	N/A	N/A
	THA	N/A	N/A	N/A	N/A

Costa et al. 2018 [24]	RHA	N/A	N/A	N/A	N/A
	THA	N/A	N/A	N/A	N/A

Tao et al. 2018 [25]	RHA	N/A	N/A	N/A	N/A
	THA	N/A	N/A	N/A	N/A

Bisseling et al. 2015 [26]	RHA	0.1 (0.1–0.1)	1.3 (1.0–1.7)	0.1 (0.1–0.1)	0.9 (0.5–1.4)
	THA	0.1 (0.1–0.1)	0.9 (0.7–1.2)	0.1 (0.1–0.1)	0.1 (0.1–0.7)

Karampinas et al. 2014 [27]	RHA	N/A	N/A	N/A	N/A
	THA	N/A	N/A	N/A	N/A

Penny et al. 2013 [28]	RHA	N/A	N/A	N/A	N/A
	THA	N/A	N/A	N/A	N/A

*Note:* All metal ion values reported in μg/L.

**FIGURE 5:**
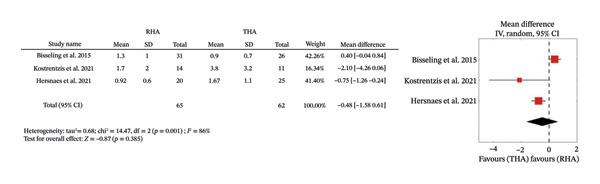
Forest plot of postoperative cobalt levels. IV = inverse variance.

**FIGURE 6:**
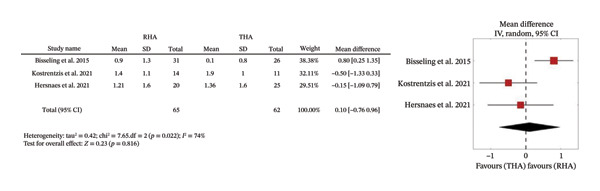
Forest plot of postoperative chromium levels. IV = inverse variance.

Both meta‐analyses comparing postoperative cobalt and chromium levels across the two groups included 3 studies, as displayed in Figures 5 and 6. The pooled MD for cobalt was 0.48 (95% CI: −1.58, 0.61; *p* = 0.385), favouring the THA group, but without statistical significance. Heterogeneity across the 3 studies for cobalt was very high with an *I* ^2^ value of 86%. The pooled MD for chromium was 0.10 (95% CI: −0.76, 0.96; *p* = 0.816), favouring the RHA group but is not statistically significant. Heterogeneity across the 3 studies for chromium was high with an *I* ^2^ value of 74%.

### 3.7. Complications, Revision Rates and Survivorship

#### 3.7.1. Complications

##### 3.7.1.1. Total

Complications were documented in 10 studies [19–28] of which 1 reported 0 complications across both groups [27]. The total number of complications was 96, with 49 (9.7%) in the RHA group compared to 47 (9.2%) in the THA group. The most commonly documented complication in the RHA group was aseptic loosening (12 cases), second was fracture (4 cases), then heterotopic ossification (4 cases) and adverse local tissue reaction (3 cases). The most frequently reported complication in the THA group was dislocation (14 cases), then followed by fracture (7 cases), aseptic loosening (5 cases), ARMD (5 cases) and infection (5 cases). A summary of all complications is provided in Table 7.

**TABLE 7:** Summary of all the complications, revision rates and survivorship.

Author/year	Treatment type	Complications	Revision rates	Survivorship
Calkins et al. 2021 [18]	RHA	N/A	N/A	N/A
	THA	N/A	N/A	N/A

LeBrun et al. 2021 [19]	RHA	0	0%	81.3% at 5 years
	THA	Squeaking or ceramic liner fracture = 2 + 1 with no revision Recurrent anterior Instability = 1	9.0%	69.7% at 5 years

Hersnaes et al. 2021 [20]	RHA	Osteolysis due to septic loosening = 1 Femoral neck fracture = 2 Aseptic loosening = 1 Unexplained hip pain without joint failure = 1 ARMD = 1	16.7%	N/A
	THA	0	0.0%	N/A

Konan et al. 2021 [21]	RHA	Aseptic loosening = 1	2.1%	98.0% (mean survival 9.9 years, 95% CI 9.8–10 years)
	THA	Pseudotumour = 1 Painful MoM with no Pseudotumour on histology = 1 Aseptic loosening = 5	12.5%	87.3% (mean survival 9.4 years, 95% CI 9–9.9)

Kostretzis et al. 2021 [22]	RHA	Femoral component loosening = 2	8.3%	N/A
	THA	Deep infection = 1 ARMD = 4	20.8%	N/A

Vendittoli et al. 2020 [23]	RHA	ARMD = 2 Femoral head loosening = 7 Acetabular fracture = 2 DVT = 1 Neuropraxia = 1 Femoroacetabular impingement = 2 Heterotopic ossification = 4	9.2%	89.2% (CI 82.3%–96.1%) at 15 years
	THA	Infections = 3 Dislocations = 4 ARMD = 1 Proximal femoral fracture = 4 DVT = 3 Neuropraxia = 2 Recurrent dislocation = 2 Periprosthetic fracture = 1	5.2%	94.2% (CI 89.3%–99.1%)

Costa et al. 2018 [24]	RHA	Revision = 1, Aspiration of hip joint = 1	1.7%	N/A
	THA	Revision = 3 Hip dislocation = 2	4.8%	N/A

Tao et al. 2018 [25]	RHA	0	0.0%	N/A
	THA	Unexplained pain = 1	2.5%	N/A

Bisseling et al. 2015 [26]	RHA	Pseudotumour = 2 Aseptic loosening due to avascular necrosis = 1	7.9%	89.5%
	THA	Recurrent dislocation = 3 Pseudotumour = 1	9.1%	90.9%

Karampinas et al. 2014 [27]	RHA	0	0.0%	N/A
	THA	0	0.0%	N/A

Penny et al. 2013 [28]	RHA	Cup displacement with dislocation = 1 Infection = 1	5.0%	N/A
	THA	Dislocation = 3	0.0%	N/A
	LHTHA	DVT/PE = 1 Pain + Increased metal ions = 1	2.0%	N/A

**FIGURE 7:**
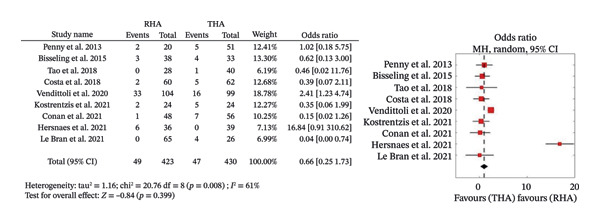
Forest plot of total number of complications. MH = Mantel–Haenszel.

Nine studies were included in the meta‐analysis comparing the difference between the total complications between RHA and THA groups, evident in Figure 7. The pooled OR was 0.66 (95% CI: 0.25, 1.73; *p* = 0.399), revealing a higher rate of complications in the THA group, but without statistical significance, evident by the *p* value. Heterogeneity across the 9 studies was high with an *I* ^2^ value of 61%.

##### 3.7.1.2. Fractures

**FIGURE 8:**
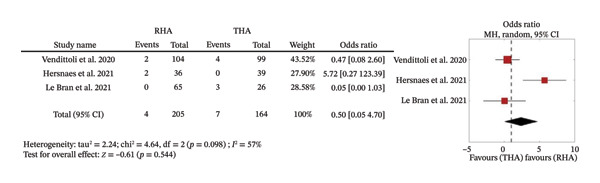
Forest plot of fractures. MH = Mantel–Haenszel.

The rate of fractures was assessed with a meta‐analysis of 3 studies, as shown in Figure 8. The pooled OR was 0.50 (95% CI: 0.05, 4.70; *p* = 0.544), showing a higher fracture rate in the THA group, but without statistical significance. Heterogeneity across the 3 studies was high with an *I* ^2^ value of 57%.

##### 3.7.1.3. Dislocations

**FIGURE 9:**
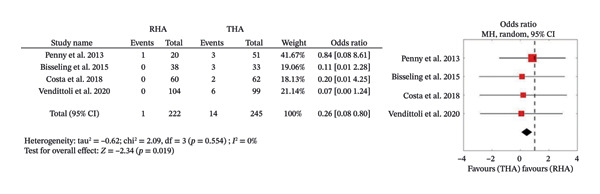
Forest plot of dislocations. MH = Mantel–Haenszel.

The difference in the rate of dislocations was assessed through 4 studies, as portrayed in Figure 9. The pooled OR was 0.26 (95% CI: 0.08, 0.80; *p* = 0.019), showing a higher rate of dislocations in the THA group, which is statistically significant. There was no heterogeneity across the 4 studies evident by the *I* ^2^ value of 0%.

#### 3.7.2. ARMD

**FIGURE 10:**
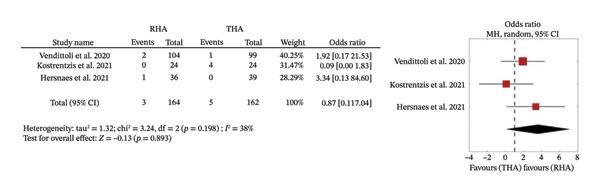
Forest plot of ARMD. MH = Mantel–Haenszel.

Three studies were included in the meta‐analysis for the difference in the rates of ARMD between the two groups, displayed in Figure 10. The pooled OR was 0.87 (95% CI: 0.11, 7.04; *p* = 0.893), showing higher rates of ARMD in the THA group, but without statistical significance. Heterogeneity across the 3 studies was low with an *I* ^2^ value of 38%.

#### 3.7.3. Aseptic Loosening

**FIGURE 11:**
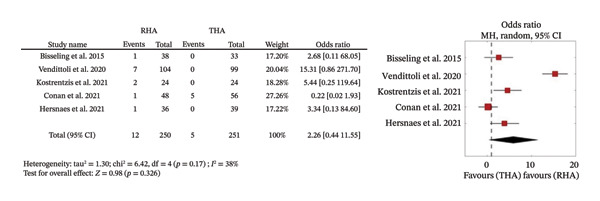
Forest plot of aseptic loosening. MH = Mantel–Haenszel.

Five studies were included in the meta‐analysis for the difference in the rates of aseptic loosening between RHA and THA, as shown in Figure 11. The pooled OR was 2.26 (95% CI: 0.44, 11.55; *p* = 0.326), showing more cases of aseptic loosening in the RHA group, but without statistical significance. Heterogeneity across the 5 studies was low with an *I* ^2^ value of 38%.

#### 3.7.4. Revision Rates

**FIGURE 12:**
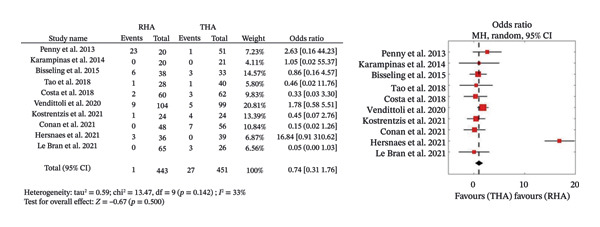
Forest plot of revision rates. MH = Mantel–Haenszel.

The revision rate was reported in 10 studies for RHA and THA [19–28], as presented in Figure 12. The total number of revisions in the RHA group was 430 (3.6%) compared to 27 (6.0%) in the THA group. The meta‐analysis for the difference in revision rates between the two groups included 10 studies. The pooled OR was 0.74 (95% CI: 0.31, 1.76; *p* = 0.142). This shows a greater revision rate for the THA group; however, there is no statistical significance. Heterogeneity across the 10 studies was low with an *I* ^2^ value of 33%.

#### 3.7.5. Survivorship

Survivorship was documented in 4 studies for both RHA and THA [19, 21, 23, 26], as shown in Table 7. The mean survivorship in the RHA group was 89.5% at a mean follow‐up of 10.0 years. The mean survivorship in the THA group was 85.5% at a mean follow‐up of 8.6 years. Although the mean survivorship across the studies favours the RHA group, none of the studies reported statistical significance between the difference in survivorship between the two groups.

### 3.8. Satisfaction and Quality of Life

Satisfaction and quality of life were reported in 4 [18, 21, 23, 26] and 2 [24, 28] studies, respectively, with a variety of methods. In the study by Calkins et al. [18], preference and satisfaction were assessed using an 8‐question survey. 30.6% preferred THA compared to 29% for RHA, while 40.4% had no preference (*p* = 0.844). Similar preference rates across the two groups are conveyed through these results, without statistical significance. However, when the patients were asked to choose only one treatment, 66.1% picked RHA, which was statistically significant (*p* ≤ 0.001). The study by Konan et al. [21] examined satisfaction rates using a mean patient satisfaction score, showing 87.8 and 83.9 in RHA and THA, respectively, favouring the RHA group. Furthermore, the proportion of patients perceiving their hip as natural was obtained by Vendittoli et al. [23]. The results showed that 60% of the RHA patients perceived their hip as natural, compared to 44% in the THA group. The VAS satisfaction score was utilised by Bisseling et al. [26], and it was reported that RHA patients were significantly more satisfied after 12 and 24 months, but no statistical significance was observed after 36 months. The VAS satisfaction score at 12 months was 92 in the RHA group versus 86 in the THA group (*p* = 0.025). At 24 months, both scores increased to 94 and 88 for RHA and THA, respectively (*p* = 0.019). The quality of life was assessed using the EQ‐5D score. Higher scores in the RHA group were reported by Costa et al. [24], but without statistical significance (*p* = 0.501). It was also shown that the score declined in both groups at a similar rate (*p* = 0.236). In the study by Penny et al. [28], the EQ‐5D scores of 0.8 for the RHA group compared to 0.9 for the THA group were reported, favouring the THA group with a statistically significant difference (*p* ≤ 0.05). A summary of the preference, satisfaction rates, perception and quality of life is provided in Table 8.

**TABLE 8:** Summary of the preference, satisfaction rates, perception and quality of life.

Author/year	Preference/satisfaction/perception	Quality of life
Calcins et al. 2021 [18]	Preference and satisfaction was assessed using an 8‐question survey (*p* = 0.844): 18 patients preferred RHA (29.0%) 19 patients preferred THA (30.6%) 25 patients (40.4%) with no preference When patients were asked to choose only 1 treatment: 41 patients chose RHA (66.1%) (*p* < 0.001) Very satisfied: RHA = 64.5% THA = 53.2% (*p* = 0.504) Delighted to spend the rest of their life with RHA = 72.6% vs THA = 62.9% (*p* = 0.295) Proportion of patients definitely recommending RHA or THA to someone else was similar (RHA = 82.4% vs THA = 75.8%) (*p* = 0.097)	N/A

LeBrun et al. 2021 [19]	N/A	N/A

Hersnaes et al. 2021 [20]	N/A	N/A

Konan et al. 2021 [21]	Mean patient satisfaction score: RHA = 87.8 (19.2) THA = 83.9 (23.9)	N/A

Kostretzis et al. 2021 [22]	N/A	N/A

Vendittoli et al. 2020 [23]	Perceived their hip as natural in the last follow‐up (*p* = 0.078): RHA = 60.0% THA = 44.0%	N/A

Costa et al. 2018 [24]	N/A	Assessed using the EQ‐5D score Higher scores in the RHA group however not statistically significant difference (*p* = 0.501) EQ‐5D score declined in both groups with no statistically significant difference in the rate of decline between the groups (*p* = 0.236)

Tao et al. 2018 [25]	N/A	N/A

Bisseling et al. 2015 [26]	Assessed using the VAS satisfaction score At 12 months (*p* = 0.025): RHA = 92, THA = 86 At 24 months (*p* = 0.019): RHA = 94 , THA = 88 No statistical significance after 36 months	N/A

Karampinas et al. 2014 [27]	N/A	N/A

Penny et al. 2013 [28]	N/A	Assessed using the EQ‐5D score RHA group = 0.8 (0.3) THA group = 0.9 (0.2) (*p* ≤ 0.05)

### 3.9. Subgroup and Sensitivity Analyses

A subgroup analysis based on the THA bearing surface was not feasible due to the limited number of studies including non‐MoM THA and the presence of mixed bearing types without separate outcome reporting. Age‐based subgroup analysis was also not feasible due to the narrow distribution of mean ages and lack of individual patient data. In addition, formal subgroup analyses based on study design, THA femoral head size and mean follow‐up duration were not feasible due to insufficiently stratified outcome data reported within the included studies. Similarly, neither sex‐based subgroup nor sensitivity analysis could be performed because outcomes were not reported separately for male and female patients, and individual patient‐level data were unavailable. However, four separate sensitivity analyses were performed to assess the robustness of the findings.

The first sensitivity analysis excluded studies with substantially younger patient populations to assess the impact of age on pooled results for UCLA, HHS, total complications, fractures and revision rates. The results of this sensitivity analysis are summarised in Table 9, and forest plots are provided in Supporting Appendix S2.1, S2.2, S2.4-6. Exclusion of the studies by LeBrun et al. [19] and Tao et al. [25] did not significantly alter the pooled effect estimates, statistical significance or overall direction of the findings. Functional outcomes for UCLA and HHS continued to favour RHA, while complication and revision outcomes remained largely unchanged. Heterogeneity for HHS was reduced in the sensitivity analysis, as shown in Table 9, although the pooled result remained nonsignificant. For fractures, there was a shift in the direction of the pooled estimate towards a higher fracture rate in the RHA group, although this also remained nonsignificant.

**TABLE 9:** Summary of the results of main meta‐analysis and sensitivity analysis.

Outcome	Analysis	Studies (*n*)	Pooled estimate	95% CI	Heterogeneity *I* ^2^ (%)	*p* value
UCLA	Main analysis	8	0.44	−0.02–0.90	78.0	0.055
	Excluding younger patients	6	0.39	−0.09–0.86	72.7	0.109
	Excluding observational studies	6	0.39	−0.09–0.86	72.7	0.109
	Excluding studies with mean follow‐up < 5 years	4	0.16	−0.30–0.62	22.2	0.498
	Excluding studies predominantly utilising smaller THA femoral head sizes (28–32 mm)	5	0.42	−0.13–1.03	69.0	0.128

HHS	Main analysis	7	1.54	−0.06–3.14	52.0	0.061
	Excluding younger patients	5	1.39	−0.02–2.80	10.9	0.053
	Excluding observational studies	4	1.05	−0.35–2.45	0.0	0.142
	Excluding studies with mean follow‐up < 5 years	3	0.46	−1.23–2.15	51.1	0.594
	Excluding studies predominantly utilising smaller THA femoral head sizes (28–32 mm)	5	0.87	−0.49–2.24	38.7	0.211

WOMAC	Main analysis	5	−1.84	−2.69–1.00	22.0	0.179
	Excluding younger patients	N/A	N/A	N/A	N/A	N/A
	Excluding observational studies	N/A	N/A	N/A	N/A	N/A
	Excluding studies with mean follow‐up < 5 years	3	−2.78	−7.19–1.63	55.7	0.216
	Excluding studies predominantly utilising smaller THA femoral head sizes (28–32 mm)	4	−2.08	−7.29–3.13	41.4	0.434

Cobalt	Main analysis	3	−0.48	−1.58–0.61	86.0	0.385
	Excluding younger patients	N/A	N/A	N/A	N/A	N/A
	Excluding observational studies	N/A	N/A	N/A	N/A	N/A
	Excluding studies with mean follow‐up < 5 years	2	−1.00	−2.03–0.03	29.6	0.056
	Excluding studies predominantly utilising smaller THA femoral head sizes (28–32 mm)	2	−1.00	−2.03–0.03	29.6	0.056

Chromium	Main analysis	3	0.10	−0.76–0.96	74.0	0.816
	Excluding younger patients	N/A	N/A	N/A	N/A	N/A
	Excluding observational studies	N/A	N/A	N/A	N/A	N/A
	Excluding studies with mean follow‐up < 5 years	2	−1.00	−2.03–0.03	29.6	0.056
	Excluding studies predominantly utilising smaller THA femoral head sizes (28–32 mm)	2	−1.00	−2.03–0.03	29.6	0.056

Total Complications	Main analysis	9	0.66	0.25–1.73	61.0	0.399
	Excluding younger patients	7	0.86	0.33–2.25	60.5	0.758
	Excluding observational studies	7	0.86	0.33–2.25	60.5	0.758
	Excluding studies with mean follow‐up < 5 years	6	0.82	0.24–2.87	65.8	0.760
	Excluding studies predominantly utilising smaller THA femoral head sizes (28–32 mm)	6	0.60	0.21–1.73	34.7	0.345

Fractures	Main analysis	3	0.50	0.05–4.70	57.0	0.544
	Excluding younger patients	2	1.17	0.11–12.48	48.8	0.898
	Excluding observational studies	2	1.17	0.11–12.48	48.8	0.898
	Excluding studies with mean follow‐up < 5 years	2	1.17	0.11–12.48	48.8	0.898
	Excluding studies predominantly utilising smaller THA femoral head sizes (28–32 mm)	N/A	N/A	N/A	N/A	N/A

Dislocations	Main analysis	4	0.26	0.08–0.80	0.0	0.019
	Excluding younger patients	N/A	N/A	N/A	N/A	N/A
	Excluding observational studies	N/A	N/A	N/A	N/A	N/A
	Excluding studies with mean follow‐up < 5 years	2	0.11	0.01–0.93	0.0	0.043
	Excluding studies predominantly utilising smaller THA femoral head sizes (28–32 mm)	2	0.50	0.08–3.16	0.0	0.460

ARMD	Main analysis	3	0.87	0.11–7.04	38.0	0.893
	Excluding younger patients	N/A	N/A	N/A	N/A	N/A
	Excluding observational studies	N/A	N/A	N/A	N/A	N/A
	Excluding studies with mean follow‐up < 5 years	N/A	N/A	N/A	N/A	N/A
	Excluding studies predominantly utilising smaller THA femoral head sizes (28–32 mm)	2	0.53	0.02–17.55	60.8	0.720

Aseptic Loosening	Main analysis	5	2.26	0.44–11.55	38.0	0.326
	Excluding younger patients	N/A	N/A	N/A	N/A	N/A
	Excluding observational studies	N/A	N/A	N/A	N/A	N/A
	Excluding studies with mean follow‐up < 5 years	4	2.33	0.30–18.05	52.9	0.417
	Excluding studies predominantly utilising smaller THA femoral head sizes (28–32 mm)	3	1.21	0.14–10.41	43.9	0.864

Revision	Main analysis	10	0.74	0.31–1.76	33	0.500
	Excluding younger patients	8	0.93	0.36–2.39	38.6	0.884
	Excluding observational studies	8	0.93	0.36–2.39	38.6	0.884
	Excluding studies with mean follow‐up < 5 years	6	0.79	0.25–2.52	47.1	0.688
	Excluding studies predominantly utilising smaller THA femoral head sizes (28–32 mm)	7	0.71	0.20–2.54	38.1	0.602

The second sensitivity analysis excluded observational studies to evaluate the influence of study design on the same outcomes, as shown in Table 9. Forest plots are provided in Supporting Appendix S2.1, S2.23-6—Exclusion of the three observational studies, by Calcins et al. [18], LeBrun et al. [19] and Tao et al. [25], similarly did not significantly alter the pooled estimates, direction of effect or statistical significance of the results. UCLA and HHS outcomes continued to favour RHA, while total complications and revision rates remained directionally consistent with the main analysis. Heterogeneity for HHS was again reduced following the exclusion of observational studies, despite remaining statistically nonsignificant. As with age‐based sensitivity analysis, fracture outcomes demonstrated a directional shift towards higher rates in the RHA group, although without statistical significance.

Additional sensitivity analyses excluding studies with a mean follow‐up of less than 5 years [19, 26–28] and studies predominantly utilising smaller THA femoral head sizes (28–32 mm) [19, 23, 26] also demonstrated largely consistent findings, which is evident in Table 9. Forest plots are provided for both sensitivity analyses in Supporting Appendix S2.7-16 and S2.17-24, respectively. For UCLA outcomes, the exclusion of shorter follow‐up studies substantially reduced heterogeneity while maintaining a pooled estimate favouring RHA. In both sensitivity analyses, HHS and WOMAC outcomes maintained the same direction of pooled results as in the main analysis, without reaching statistical significance. Cobalt and chromium ion analyses remained nonsignificant as well, although heterogeneity was reduced following exclusion of shorter follow‐up studies and smaller femoral head size studies. With respect to complications, the pooled estimates for total complications, ARMD, aseptic loosening and revision rates remained directionally consistent across all sensitivity analyses. Importantly, the significantly lower dislocation risk observed in RHA in the main analysis remained significant after the exclusion of studies with mean follow‐up of less than 5 years, with the direction of effect continuing to favour RHA. However, statistical significance was lost after exclusion of studies predominantly utilising smaller THA femoral head sizes, although the pooled estimate still remained in favour of RHA. Forest plots for all sensitivity analyses are provided in Supporting Appendix S2.

### 3.10. GRADE Assessment of Certainty of Evidence

The results for the GRADE assessment of certainty of evidence are displayed in Table 10. Moreover, detailed reasons for downgrade and outcome‐specific GRADE judgements and explanations are provided in Supporting Appendix S3.

**TABLE 10:** Summary of findings of GRADE assessment.

**Comparative outcomes of resurfacing vs total hip arthroplasty**						

Patient or population: adults undergoing hip arthroplasty for hip joint disease and eligible for resurfacing hip arthroplasty or total hip arthroplasty						
Settings: secondary care/orthopaedic arthroplasty centres						
Intervention: resurfacing hip arthroplasty (RHA)						
Comparison: total hip arthroplasty (THA)						

**Outcome**	**Comparator values and anticipated absolute effects**	**Relative or mean effect**	**No. of studies**	**Certainty of evidence**	**Reason for downgrade**	**Interpretation**

UCLA	Mean postoperative UCLA: 6.9 with THA; RHA was 0.44 points higher	MD 0.44, 95% CI ‐0.02 to 0.90	8	Low ⊕⊕◯◯	Inconsistency, *I* ^2^ = 78%; imprecision, CI crosses no effect	RHA may slightly improve UCLA activity score, but the evidence is uncertain.
HHS	Mean postoperative HHS: 92.4 with THA; RHA was 1.54 points higher	MD 1.54, 95% CI ‐0.06 to 3.14	7	Moderate ⊕⊕⊕◯	Imprecision, CI crosses no effect; Inconsistency was considered but not downgraded because sensitivity analyses explained much of the heterogeneity	HHS is probably similar between groups, although the point estimate slightly favours RHA.
WOMAC	Mean postoperative WOMAC: 91.7 with THA; RHA was 1.84 points lower	MD ‐1.84, 95% CI ‐2.69 to 1.00	5	Moderate ⊕⊕⊕◯	Imprecision, CI crosses no effect	WOMAC is probably similar between groups. Point estimate favours THA.
Cobalt ion levels	Mean postoperative cobalt: 2.12 μg/L with THA; RHA was 0.48 μg/L lower	MD ‐0.48 μg/L, 95% CI ‐1.58 to 0.61	3	Low ⊕⊕◯◯	Inconsistency, *I* ^2^ = 86%; imprecision, CI crosses no effect	Cobalt levels may be similar, but the evidence is uncertain.
Chromium ion levels	Mean postoperative chromium: 1.12 μg/L with THA; RHA was 0.10 μg/L higher	MD 0.10 μg/L, 95% CI ‐0.76 to 0.96	3	Low ⊕⊕◯◯	Inconsistency, *I* ^2^ = 74%; imprecision, CI crosses no effect	Chromium levels may be similar, but the evidence is uncertain.
Total complications	Risk with THA: 92 per 1000; corresponding risk with RHA: 63 per 1000, 95% CI 25 to 149 per 1000	OR 0.66, 95% CI 0.25 to 1.73	9	Low ⊕⊕◯◯	Inconsistency, *I* ^2^ = 61%; imprecision, CI includes benefit and harm	Overall complications may be similar between RHA and THA.
Fractures	Risk with THA: 14 per 1000; corresponding risk with RHA: 7 per 1000, 95% CI 1 to 63 per 1000	OR 0.50, 95% CI 0.05 to 4.70	3	Very low ⊕◯◯◯	Very serious imprecision, very wide CI; inconsistency, *I* ^2^ = 57%	Evidence is very uncertain for fracture risk.
Dislocations	Risk with THA: 27 per 1000; corresponding risk with RHA: 7 per 1000, 95% CI 2 to 22 per 1000	OR 0.26, 95% CI 0.08 to 0.80	4	Moderate ⊕⊕⊕◯	Indirectness/applicability due to THA head size and bearing surface variation	RHA probably reduces dislocation risk compared with THA.
ARMD	Risk with THA: 10 per 1000; corresponding risk with RHA: 9 per 1000, 95% CI 1 to 66 per 1000	OR 0.87, 95% CI 0.11 to 7.04	3	Low ⊕⊕◯◯	Very serious imprecision, very wide CI	ARMD risk may be similar, but the evidence is uncertain.
Aseptic loosening	Risk with THA: 10 per 1000; corresponding risk with RHA: 22 per 1000, 95% CI 4 to 104 per 1000	OR 2.26, 95% CI 0.44 to 11.55	5	Low ⊕⊕◯◯	Very serious imprecision, very wide CI	RHA may increase aseptic loosening, but the evidence is uncertain.
Revision rates	Risk with THA: 60 per 1000; corresponding risk with RHA: 45 per 1000, 95% CI 19 to 101 per 1000	OR 0.74, 95% CI 0.31 to 1.76	10	Moderate ⊕⊕⊕◯	Imprecision, CI includes benefit and harm	Revision rates are probably similar between RHA and THA.
Survivorship	Mean survivorship: 85.5% with THA and 89.5% with RHA	Not pooled	4	Low ⊕⊕◯◯	Indirectness from different follow‐up durations; imprecision, no pooled CI	Survivorship may be similar between groups, but the comparison is limited by different follow‐up durations and lack of pooled estimates.
Satisfaction/quality of life	Satisfaction generally favoured RHA; quality‐of‐life findings were mixed	Not pooled	6	Low ⊕⊕◯◯	Indirectness from varied instruments; imprecision, no pooled estimate	Satisfaction may favour RHA, but findings were heterogeneous and narratively synthesised.

*Note:* Certainty of evidence was assessed using the GRADE approach. Concise reasons for downgrading are shown in the table; the detailed outcome‐specific GRADE judgements and explanations are provided in Supporting Appendix S3.

Abbreviations: ARMD, adverse reaction to metal debris; CI, confidence interval; MD, mean difference; OR, odds ratio; RHA, resurfacing hip arthroplasty; THA, total hip arthroplasty.

## 4. Discussion

This systematic review and meta‐analysis meticulously evaluate the outcomes of RHA and THA, offering a targeted assessment for younger, more active patients frequently overlooked in broader research. Despite the widespread success and growing adoption of hip arthroplasties, detailed insights into the relative effectiveness and appropriateness of RHA versus THA are essential, especially considering the varied demographics and lifestyle requirements of patients.

A refined analysis of functional outcomes and longevity for both RHA and THA is provided by this study’s findings. Notably, while RHA ostensibly supports higher functional outcomes in younger individuals, evidenced by marginally better UCLA and HHSs, the differences are not statistically significant. This suggests that while theoretical benefits in terms of range of motion and sports return may be offered by RHA, the clinical implications are subtle and do not universally justify the choice over THA. A marginal numeric difference in function alone is insufficient to recommend RHA; instead, functional expectations should be discussed with patients as likely similar for both procedures, and the choice should hinge on implant‐specific risks and patient anatomy.

Nevertheless, the analysis of complications and revision rates adds a critical layer of consideration. Although overall complication and revision rates between RHA and THA were comparable, there is a statistically significantly higher rate of dislocations in the THA group, which is a robust and critical finding of this study, representing a key differentiating outcome with direct clinical implications. Dislocation is not only a common cause of early failure but also associated with recurrent instability, need for revision surgery and reduced patient confidence and quality of life, making it a clinically decisive disadvantage of THA in this context. Accordingly, the increased dislocation risk associated with THA should be interpreted as a central trade‐off in surgical decision‐making rather than an isolated complication rate. Conversely, while the higher incidence of specific complications, such as aseptic loosening and metal ion release in the RHA group, did not reach statistical significance, it remains clinically relevant and reflects known implant‐specific risks, reinforcing the importance of appropriate patient selection and surgical expertise, as discussed in the study by Amstutz et al. [29]. The choice between THA and RHA therefore reflects a direct comparison between the statistically significantly greater dislocation risk in THA and the linked implant‐specific risks of RHA, which, although not statistically significant in this study, remain established concerns. However, this comparison should be interpreted with caution as any apparent benefit of RHA may stem from patient selection rather than implant superiority. Patients undergoing RHA are inherently preselected, typically comprising younger, predominantly male patients, with larger acetabula, as the procedure requires sufficiently large acetabular dimensions for implant suitability [30]. This introduces selection bias, which may influence the observed complication rates and limits direct comparability. Notably, sensitivity analysis excluding studies predominantly utilising smaller THA femoral head sizes resulted in the loss of statistical significance for the dislocation outcome, although the direction of effect remained unchanged, suggesting that this finding may be partially influenced by implant‐related factors and should therefore be interpreted with caution. Overall, while some outcomes appear broadly similar between RHA and THA, the markedly higher dislocation risk with THA should not be viewed as a marginal finding but rather as a central consideration in surgical planning and patient counselling.

The reduction in complication rates associated with both RHA and THA remains a pivotal aspect of improving patient outcomes. Advanced surgical precision, supported by preoperative planning with high‐resolution imaging techniques, plays a crucial role in tailoring the surgical approach to an individual’s anatomy, thus minimising risks of malpositioning and complications such as dislocation and aseptic loosening. Additionally, the selection of implant materials that minimise wear and potential metal ion release is particularly vital. This consideration is crucial for younger patients who face greater risks over extended follow‐up periods, especially with RHA involving MoM bearings, where metal ions can induce systemic and localised adverse reactions.

Implementing rigorous postoperative monitoring and customised rehabilitation protocols further aids in mitigating risks, facilitating early detection and management of issues. This approach is enhanced by the insights from Andriollo et al., who emphasise the significance of technological advancements in surgical planning and execution in reducing complication rates in hip arthroplasty [31]. It is also suggested by the literature that susceptibility to metal ion toxicity varies based on genetic and other health factors, underscoring the importance of preoperative genetic screening. As noted by Posada et al., patient eligibility for specific types of implants can be refined through such screenings and postoperative complications can be further reduced, thereby enhancing long‐term health outcomes by strategically addressing both surgical execution and patient‐specific factors [32]. Synthesis of cobalt and chromium ion data within the current meta‐analysis revealed no statistical difference in ion release between RHA and THA, while simultaneously, pooled mean ion levels reported in the included studies were found to be below commonly used action thresholds, such as 7 μg/L recommended by the Medicines and Healthcare products Regulatory Agency (MHRA) [33]. This population‐level finding is reassuring because elevated metal ions are a recognised risk factor for ARMD, periprosthetic osteolysis and, in some cases, revision surgery [34]. However, the result requires cautious interpretation since only a minority of included studies reported ion data, assays and metrices varied, and sampling time points and follow‐up durations were inconsistent across studies. For these reasons, guideline‐based surveillance should continue to be undertaken by clinicians, with results interpreted alongside symptoms and cross‐sectional imaging, and implant‐specific thresholds applied where available. When patients are counselled, particularly younger individuals considering RHA, both the reassuring aggregate ion data and the potential implications of lifelong monitoring in the event of elevated levels should be discussed.

Furthermore, through examining the impact of RHA and THA on patient satisfaction and quality of life, it is revealed by the current study that higher satisfaction rates and an improved quality of life are generally reported by patients undergoing RHA compared to those receiving THA. This enhancement is attributed to the better restoration of hip function and a reduced perception of disability, particularly significant in younger, more active patients. These findings align with those reported by Za et al., who note similar trends and underscored the importance of patient‐centred care approaches [35]. Such approaches ensure that surgical decisions are tailored not only to clinical indicators but also to the patients′ lifestyle and activity level expectations, enhancing overall postoperative satisfaction and quality of life.

This systematic review builds upon and distinctly diverges from prior studies such as the one mentioned above by Za et al., which focused broadly on various demographic groups and incorporated randomised clinical trials up to 2023 [35]. The timeframe is expanded by the current study, and the inclusion criteria are also specified to provide more comprehensive insights, particularly tailored for younger, more active patients. Updated statistical tools and NOS criteria are used in this rigorous meta‐analysis, ensuring robustness and reliability of findings. Lastly, personalised surgical options are advocated in this study, with the aim to minimise complications and optimise long‐term outcomes, thus providing a clearer pathway for clinical decision‐making and policy development in orthopaedic practices to maximise the benefits of both RHA and THA for appropriate patient populations.

The heterogeneity observed across the selected studies is likely multifactorial and has important implications for the interpretation of the pooled estimates. High heterogeneity was observed for UCLA, HHS, cobalt and chromium ion levels, total complications and fractures. For HHS, although heterogeneity was high in the main analysis, sensitivity analyses excluding younger cohorts and observational studies substantially reduced heterogeneity, suggesting that part of the variability may be explained by differences in patient population and study design. More broadly, between‐study variability may reflect differences in patient demographics, activity level, implant design, bearing surface, THA femoral head size, follow‐up duration and outcome definitions. Variable follow‐up duration is particularly relevant for implant survivorship, revision, aseptic loosening and other long‐term complications, which may not be fully captured in studies with shorter follow‐up. Similarly, implant design and femoral head size may affect joint stability and dislocation risk, while the male‐predominant demographic of the included cohorts further limits generalisability, particularly to female patients and those with smaller acetabular dimensions. For metal ion outcomes specifically, variability in bearing surfaces and femoral head sizes across THA comparators likely contributed to heterogeneity and limits the generalisability of the pooled estimates. Consequently, pooled results with substantial heterogeneity should be interpreted as average effects across clinically diverse studies rather than as uniform treatment effects applicable to all patients or implant types. In these outcomes, the direction and statistical significance of the pooled estimate should therefore be interpreted cautiously. In contrast, outcomes with low or absent heterogeneity, such as dislocations and revision rates, provide more internally consistent estimates. These concerns regarding heterogeneity and applicability are incorporated into the GRADE assessment of the certainty of evidence, discussed below.

The GRADE assessment further supports cautious interpretation of the findings. Moderate‐certainty evidence suggested that RHA is associated with a lower risk of dislocation compared with THA, while revision rates and WOMAC scores were supported by moderate‐certainty evidence and appeared broadly similar between groups. In contrast, certainty was low for UCLA, cobalt and chromium ion levels, total complications, ARMD, aseptic loosening, survivorship, and satisfaction or quality‐of‐life outcomes, and very low for fracture outcomes. Downgrading was driven mainly by inconsistency, imprecision and indirectness rather than clear methodological limitations. These findings indicate that although RHA shows favourable trends for some outcomes, most comparative results should be interpreted cautiously, with reduced dislocation risk representing the most robust comparative advantage.

While extensive, this systematic review encounters limitations that impact the interpretability and generalisability of its findings. Firstly, a degree of heterogeneity is introduced by the variability in study designs among the included studies, which complicates the aggregation and comparison of outcomes, potentially biasing the results. High heterogeneity across studies has been demonstrated in meta‐analyses for UCLA, HHS, total complications, fractures and metal ions. As previously mentioned, this may reflect differences in population size, surgical techniques, follow‐up durations and outcome definitions. Although a random‐effects model has been used in this meta‐analysis to account for between‐study variability, the findings in the aforementioned categories should be interpreted with caution given the high heterogeneity. However, sensitivity analyses demonstrated that the exclusion of studies with younger cohorts and observational designs did not materially alter the direction or significance of the pooled estimates, supporting the robustness of the main findings. Similarly, the exclusion of studies with shorter follow‐up durations and those predominantly utilising smaller THA femoral head sizes did not alter the overall conclusions; however, while the lower dislocation risk observed in RHA remained significant after excluding shorter follow‐up studies, statistical significance was lost following the exclusion of studies with smaller femoral head sizes, despite the direction of effect remaining unchanged. The inability to perform formal subgroup analyses due to limited reporting and lack of individual patient data restricts more granular interpretation. The predominance of male participants in these studies also poses significant demographic constraints, limiting the applicability of the findings to women, who constitute a substantial proportion of the patient population undergoing hip arthroplasty. Moreover, the focus of many studies on short‐ to medium‐term outcomes means that there is a lack of long‐term data, which is critical for assessing the durability and longevity of prosthetic implants, key factors in clinical decision‐making, especially for younger patients who require longer‐lasting solutions. Another notable limitation is the underrepresentation of patient‐reported outcome measures in the data, which are crucial for evaluating the impact of surgical interventions from the patients′ perspectives. Additionally, the approach of no predefined outcome measures while pragmatic introduces a degree of subjectivity in the outcome selection and a risk of selective reporting. Simultaneously, the review is restricted to studies published in English, potentially excluding relevant findings published in other languages and contributing to publication bias, where studies reporting positive outcomes are more frequently published. A further limitation of this study is the selection bias arising from anatomical constraints in candidates for RHA. In general, larger femoral heads are required in RHA; thus, in patients with smaller acetabular measurements, for instance, young females, this procedure may not be feasible [29]. RHA cohorts are likely skewed towards patients with a larger acetabulum due to this restriction, therefore introducing selection bias. This anatomical constraint helps explain why MoM designs continue to be used in RHA, as they are more appropriate for larger acetabula. Although sample sizes for the two groups are similar in this study, this may be reducing the generalisability and applicability of the findings and potentially influencing the comparative outcomes between the two procedures. Moreover, the study protocol was registered retrospectively with OSF, as prospective registration in PROSPERO had not been undertaken at the outset of the review. Although retrospective registration improves transparency and ensures public accessibility of the study protocol, it does not offer the same methodological safeguards as prospective registration. In particular, because methods were not publicly specified prior to study conduct, the possibility of selective reporting bias cannot be fully excluded. In addition, retrospective registration limits the ability to independently verify whether methodological decisions, outcome selection or analytical approaches were influenced during the review process. Collectively, these limitations highlight the need for future research to incorporate long‐term follow‐up data, involve diverse patient demographics, expand the inclusion of PROMs and account for implant size‐related eligibility constraints to enhance the robustness and clinical applicability of the findings.

## 5. Conclusion

In essence, while important insights into the comparative outcomes of RHA and THA are provided by this review, the decision on the most appropriate type of hip arthroplasty should be individualised, on the basis of comprehensive assessments of long‐term outcomes, patient‐centred metrics and personal lifestyle needs. The existing gaps in the literature should be addressed in future studies, with particular focus on long‐term data, inclusive demographic representation and the integration of broader patient‐reported outcomes.

## Funding

This research received no specific grant from any funding agency in the public, commercial or not‐for‐profit sectors.

## Conflicts of Interest

The authors declare no conflicts of interest.

## Supporting Information

Additional supporting information can be found online in the Supporting Information section.

## Supporting information


**Supporting Information** Supporting Appendix: Full search strategy and Boolean queries for all databases.

## Data Availability

The data that support the findings of this study are available from the corresponding author upon reasonable request.
