# Supplementary material for: Comparative Outcomes of Resurfacing vs Total Hip Arthroplasty: A Systematic Review and Meta‐Analysis
Source: Adv Orthop. 2026 Jul 7;2026:4661645. doi: 10.1155/aort/4661645 (PMC13341940; doi:10.1155/aort/4661645)
Supplement: Supplementary file 1 — Supporting Information Supporting Appendix: Full search strategy and Boolean queries for all databases. [file AORT-2026-4661645-s001.docx]

**Supplementary Appendix**

**Appendix S1: Boolean Queries**

**PubMed**

(("Arthroplasty, Replacement, Hip"[MeSH]

OR "total hip arthroplasty"[tiab]

OR "total hip replacement"[tiab]

OR THA[tiab]

OR THR[tiab]))

AND

(("hip resurfacing arthroplasty"[tiab]

OR "hip resurfacing"[tiab]

OR "resurfacing hip arthroplasty"[tiab]

OR "hip resurfacing replacement"[tiab]

OR HRA[tiab]

OR RHA[tiab]))

AND

((compar*[tiab]

OR versus[tiab]

OR vs[tiab]

OR outcome[tiab]

OR outcomes[tiab]))

**EMBASE**

(

'total hip arthroplasty'/exp

OR 'total hip arthroplasty':ti,ab

OR 'total hip replacement':ti,ab

OR tha:ti,ab

OR thr:ti,ab

)

AND

(

'hip resurfacing arthroplasty'/exp

OR 'hip resurfacing':ti,ab

OR 'hip resurfacing arthroplasty':ti,ab

OR 'resurfacing hip arthroplasty':ti,ab

OR 'hip resurfacing replacement':ti,ab

OR hra:ti,ab

OR rha:ti,ab

)

AND

(

compar*:ti,ab

OR versus:ti,ab

OR vs:ti,ab

OR outcome:ti,ab

OR outcomes:ti,ab

)

**Cochrane**

(("total hip arthroplasty"

OR "total hip replacement"

OR THA

OR THR):ti,ab,kw)

AND

(("hip resurfacing"

OR "hip resurfacing arthroplasty"

OR "resurfacing hip arthroplasty"

OR "hip resurfacing replacement"

OR HRA

OR RHA):ti,ab,kw)

AND

((compar*

OR versus

OR vs

OR outcome

OR outcomes):ti,ab,kw)

**Appendix S2: Sensitivity Analysis**

**Figure S2.1:** Forest plot of sensitivity analysis for UCLA score excluding studies [19, 25] due to substantially younger patient populations. An identical dataset and results were obtained in the sensitivity analysis excluding observational studies [19, 25]; therefore, a separate plot is not shown.


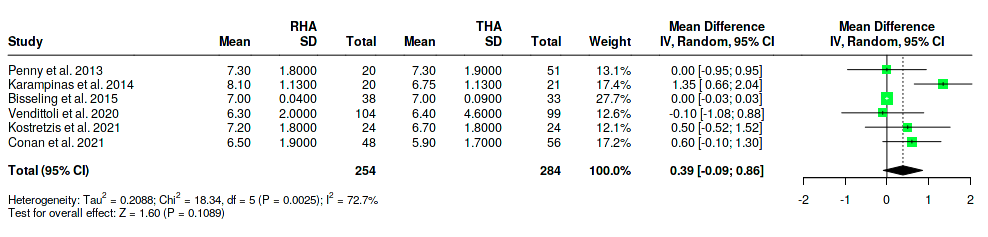


**Figure S2.2:** Forest plot of sensitivity analysis for HHS score excluding studies [19, 25] due to substantially younger patient populations.


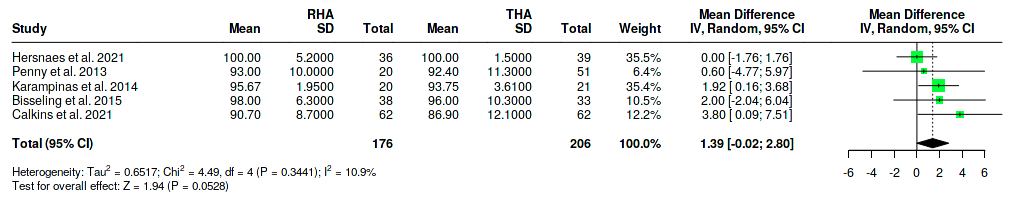


**Figure S2.3:** Forest plot of sensitivity analysis for HHS score excluding observational studies [18, 19, 25].


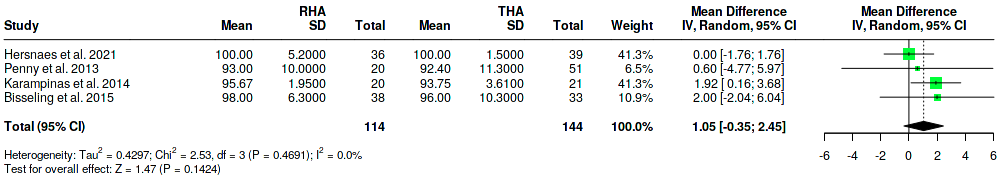


**Figure S2.4:** Forest plot of sensitivity analysis for total complications excluding studies [19, 25] due to substantially younger patient populations. An identical dataset and results were obtained in the sensitivity analysis excluding observational studies [19, 25]; therefore, a separate plot is not shown.


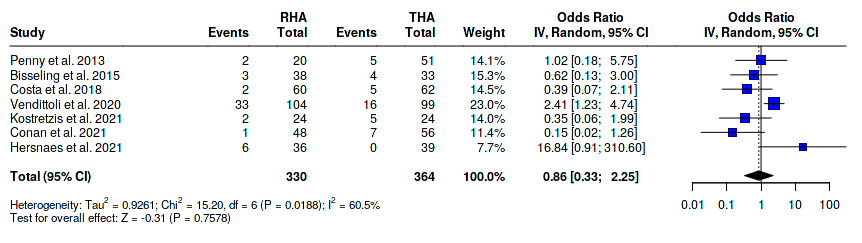


**Figure S2.5:** Forest plot of sensitivity analysis for fractures excluding the study [19] due to substantially younger patient populations. An identical dataset and results were obtained in the sensitivity analysis excluding the observational study [19]; therefore, a separate plot is not shown.


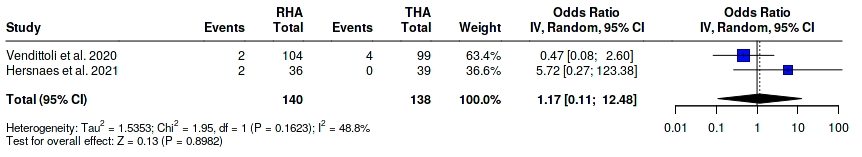


**Figure S2.6:** Forest plot of sensitivity analysis for revision excluding studies [19, 25] due to substantially younger patient populations. An identical dataset and results were obtained in the sensitivity analysis excluding observational studies [19, 25]; therefore, a separate plot is not shown.


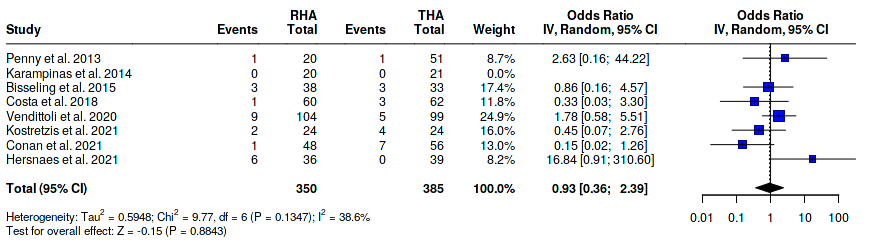


**Figure S2.7:** Forest plot of sensitivity analysis for UCLA excluding studies [19,26-28] due to mean follow-up of less than 5 years.


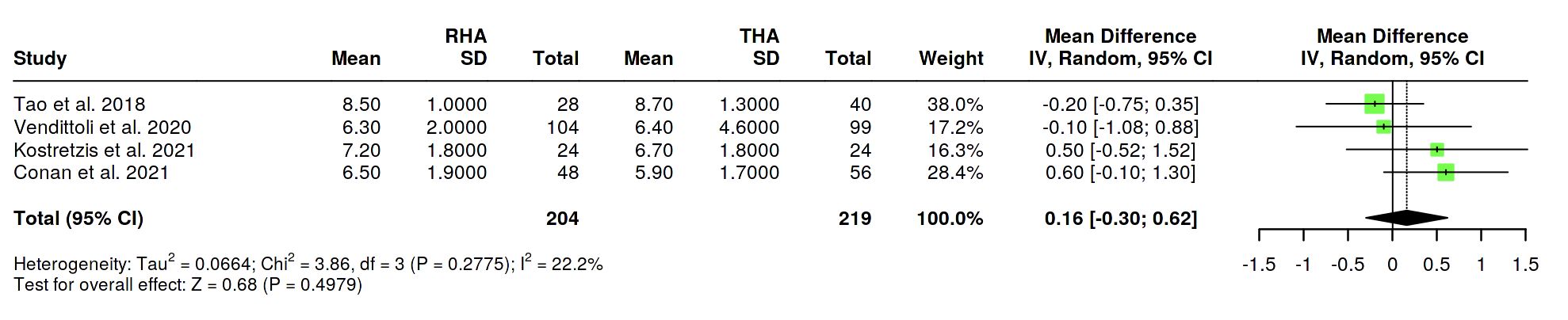


**Figure S2.8:** Forest plot of sensitivity analysis for HHS excluding studies [19,26-28] due to mean follow-up of less than 5 years.


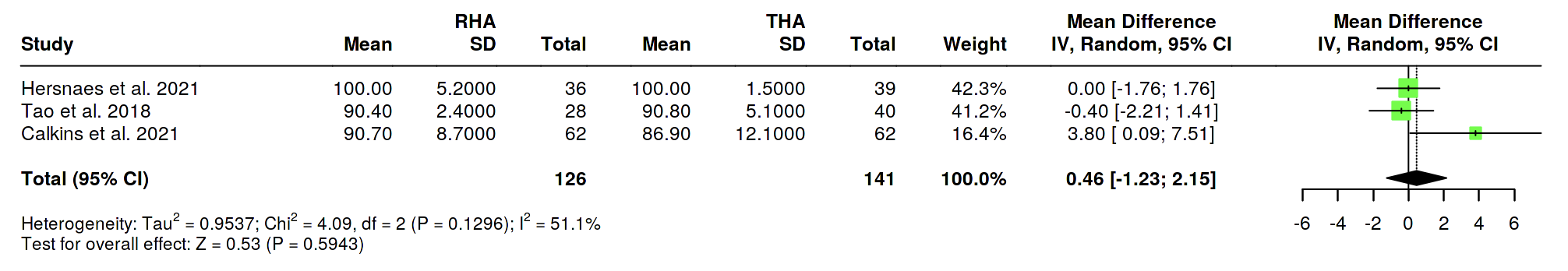


**Figure S2.9:** Forest plot of sensitivity analysis for WOMAC excluding studies [19,26-28] due to mean follow-up of less than 5 years.


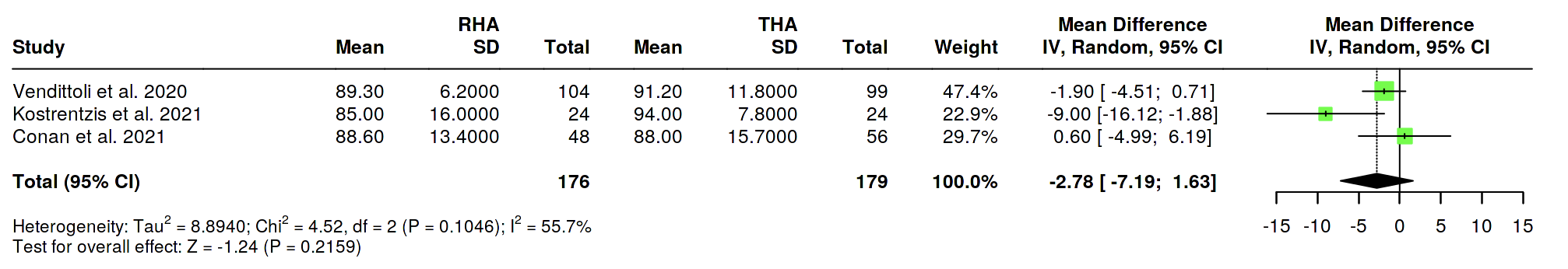


**Figure S2.10:** Forest plot of sensitivity analysis for Cobalt excluding the study [26] due to mean follow-up of less than 5 years. An identical dataset and results were obtained in the sensitivity analysis excluding the study predominantly utilizing smaller THA femoral head sizes (28-32mm) [26]; therefore, a separate plot is not shown.


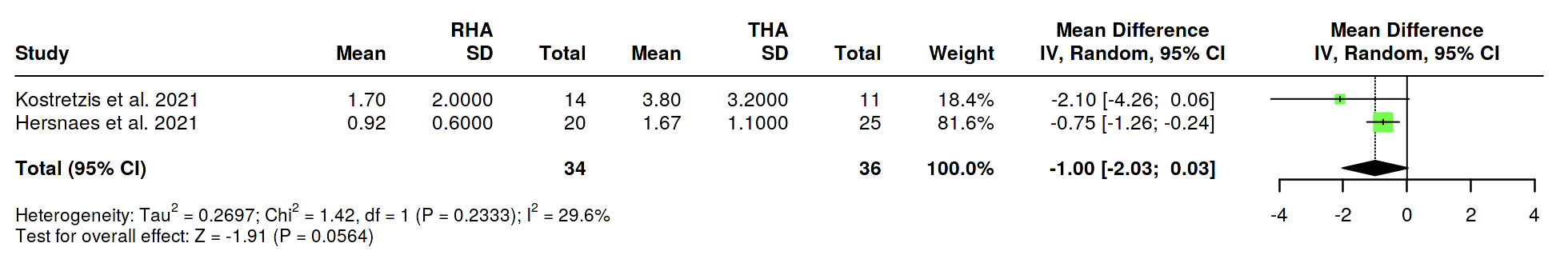


**Figure S2.11:** Forest plot of sensitivity analysis for Chromium excluding the study [26] due to mean follow-up of less than 5 years. An identical dataset and results were obtained in the sensitivity analysis excluding the study predominantly utilizing smaller THA femoral head sizes (28-32mm) [26]; therefore, a separate plot is not shown.


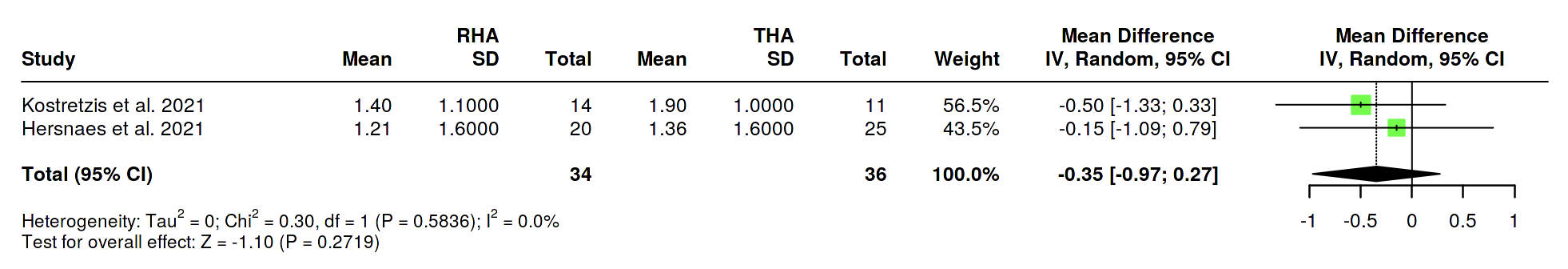


**Figure S2.12:** Forest plot of sensitivity analysis for total complications excluding studies [19,26,28] due to mean follow-up of less than 5 years.


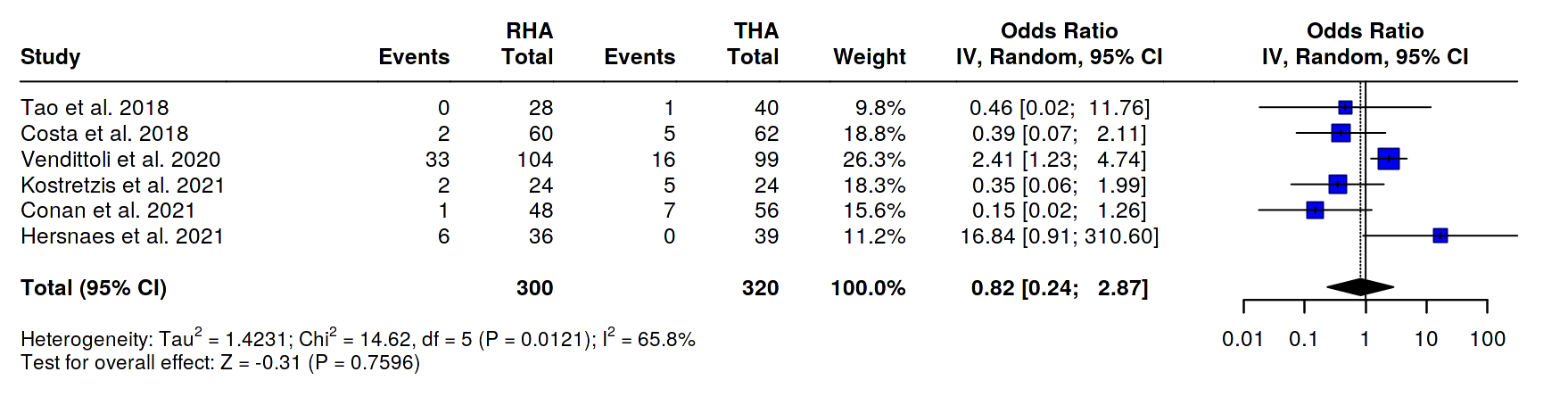


**Figure S2.13:** Forest plot of sensitivity analysis for fractures excluding the study [19] due to mean follow-up of less than 5 years.


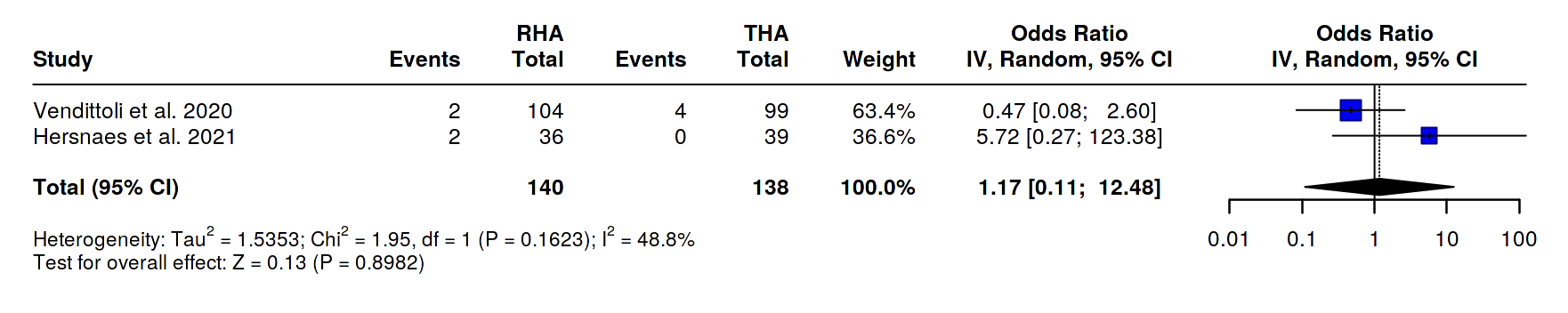


**Figure S2.14:** Forest plot of sensitivity analysis for dislocations excluding studies [26,28] due to mean follow-up of less than 5 years.


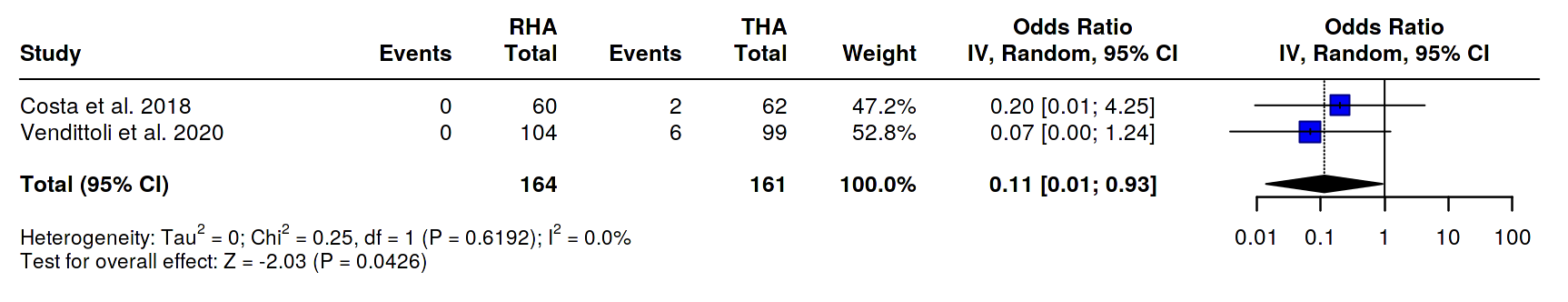


**Figure S2.15:** Forest plot of sensitivity analysis for aseptic loosening excluding the study [26] due to mean follow-up of less than 5 years.


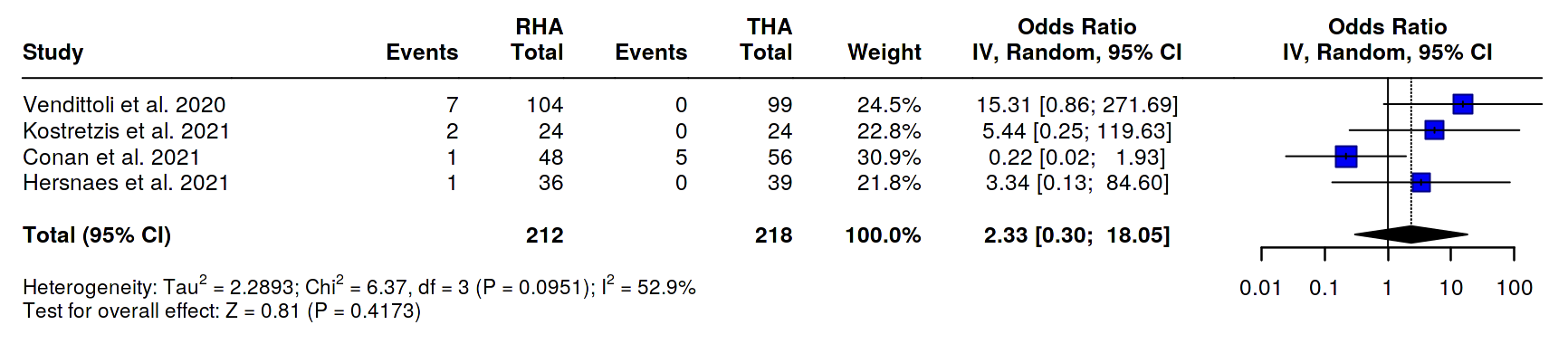


**Figure S2.16:** Forest plot of sensitivity analysis for revision rate excluding studies [19,26,28] due to mean follow-up of less than 5 years.


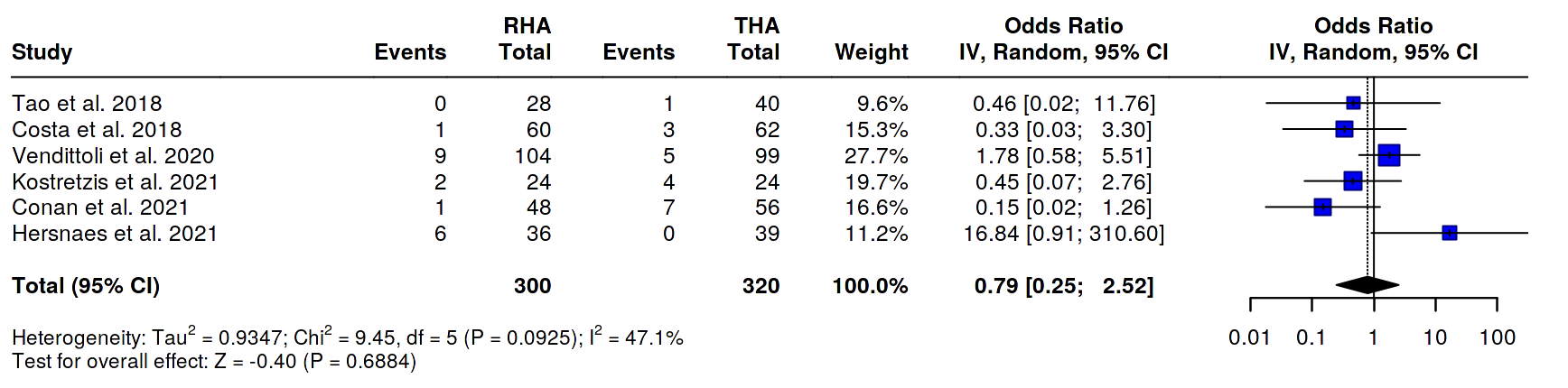


**Figure S2.17:** Forest plot of sensitivity analysis for UCLA excluding studies [19,23,26] predominantly utilizing smaller THA femoral head sizes (28-32mm).


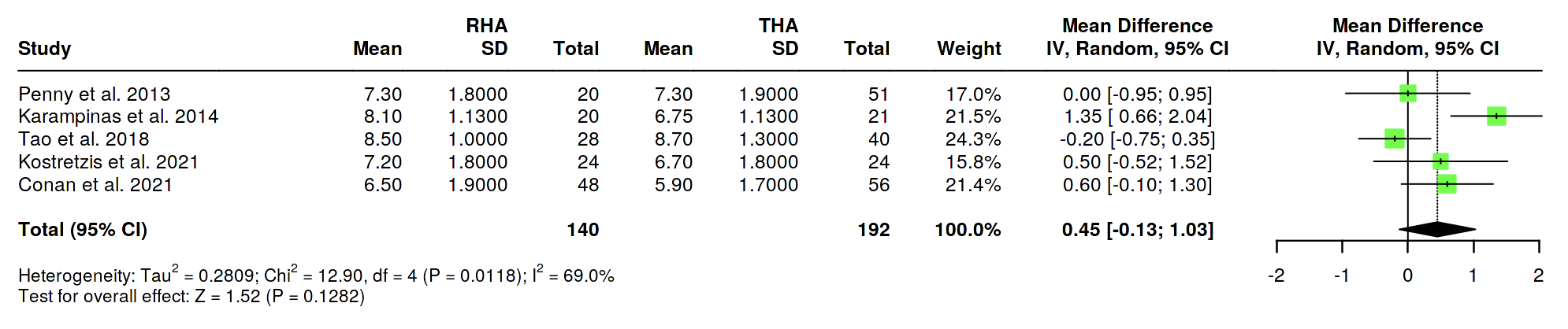


**Figure S2.18:** Forest plot of sensitivity analysis for HHS excluding studies [19,26] predominantly utilizing smaller THA femoral head sizes (28-32mm).


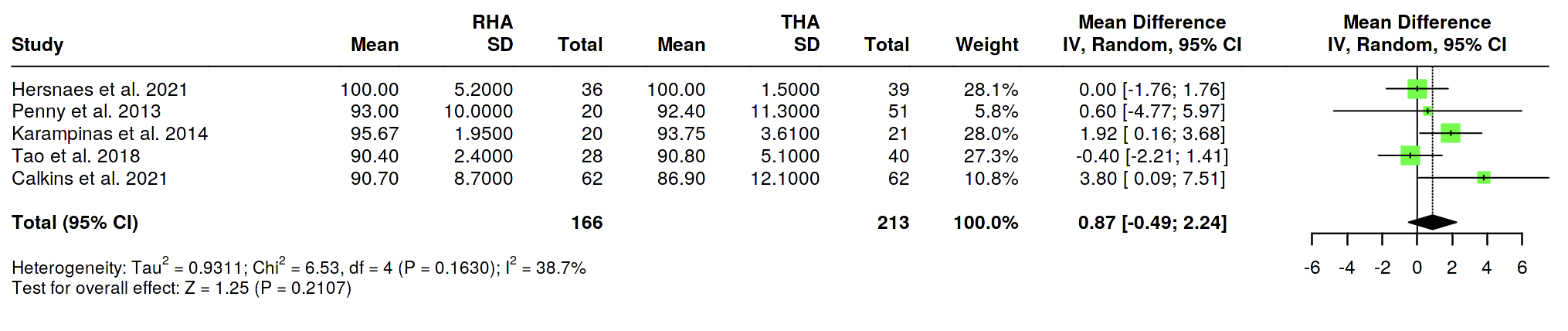


**Figure S2.19:** Forest plot of sensitivity analysis for WOMAC excluding the study [23] which is predominantly utilizing smaller THA femoral head sizes (28-32mm).


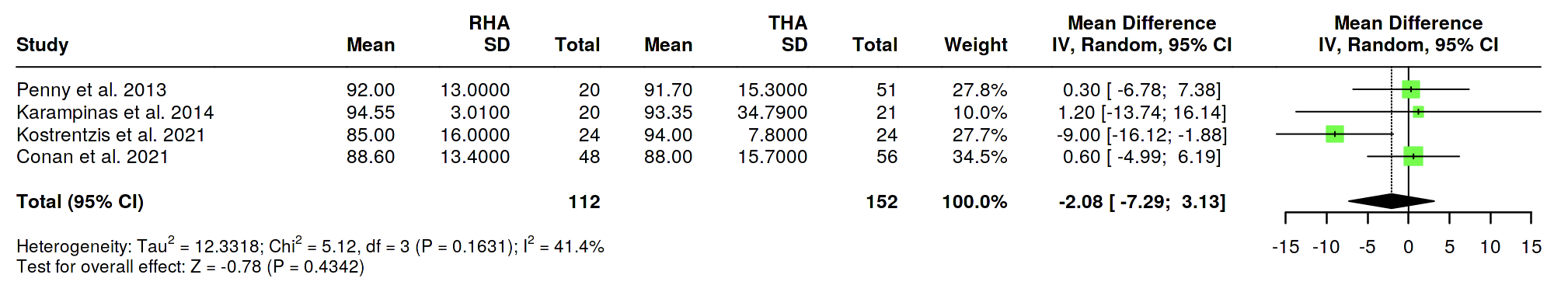


**Figure S2.20:** Forest plot of sensitivity analysis for total complications excluding studies [19,23,26] predominantly utilizing smaller THA femoral head sizes (28-32mm).


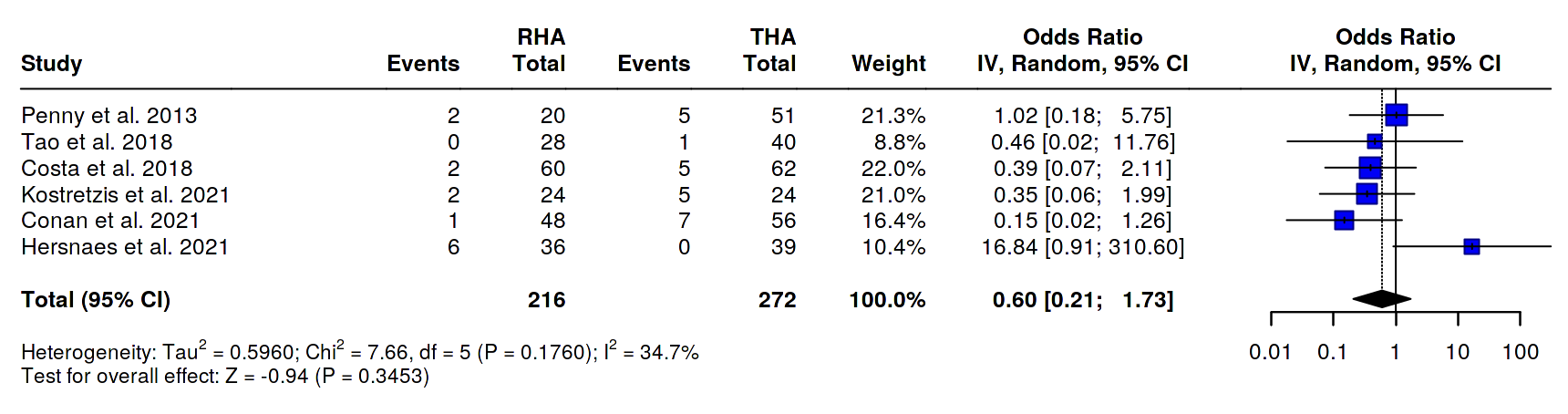


**Figure S2.21:** Forest plot of sensitivity analysis for dislocations excluding studies [23,26] predominantly utilizing smaller THA femoral head sizes (28-32mm).


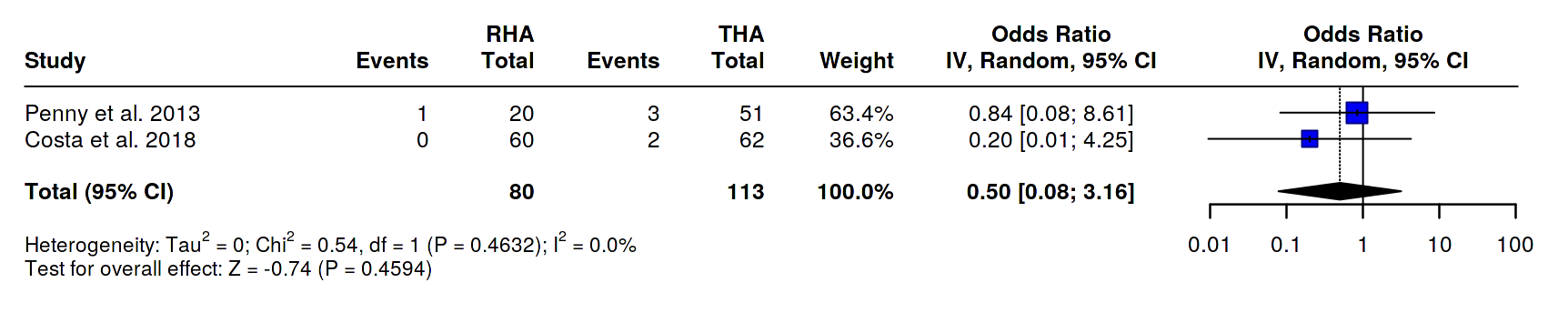


**Figure S2.22:** Forest plot of sensitivity analysis for ARMD excluding the study [23] which is predominantly utilizing smaller THA femoral head sizes (28-32mm).


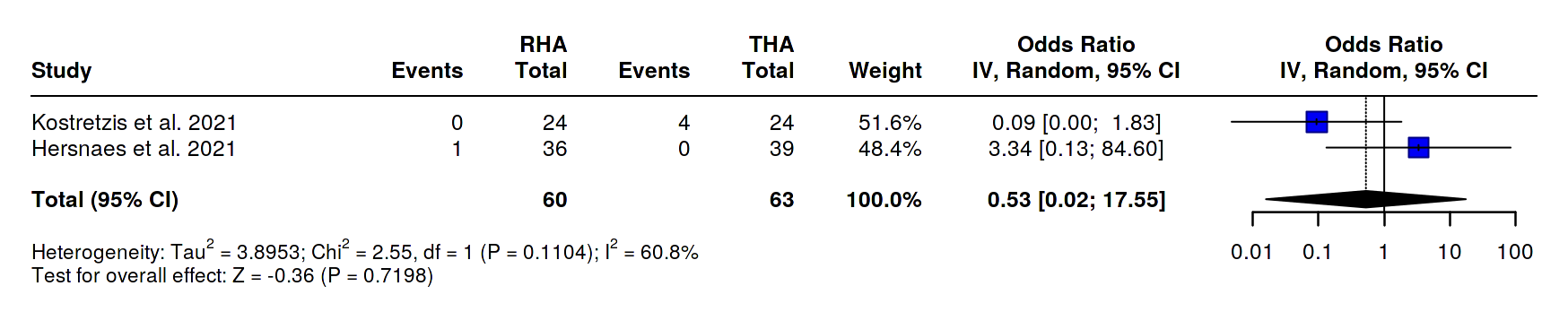


**Figure S2.23:** Forest plot of sensitivity analysis for aseptic loosening excluding studies [23,26] predominantly utilizing smaller THA femoral head sizes (28-32mm).


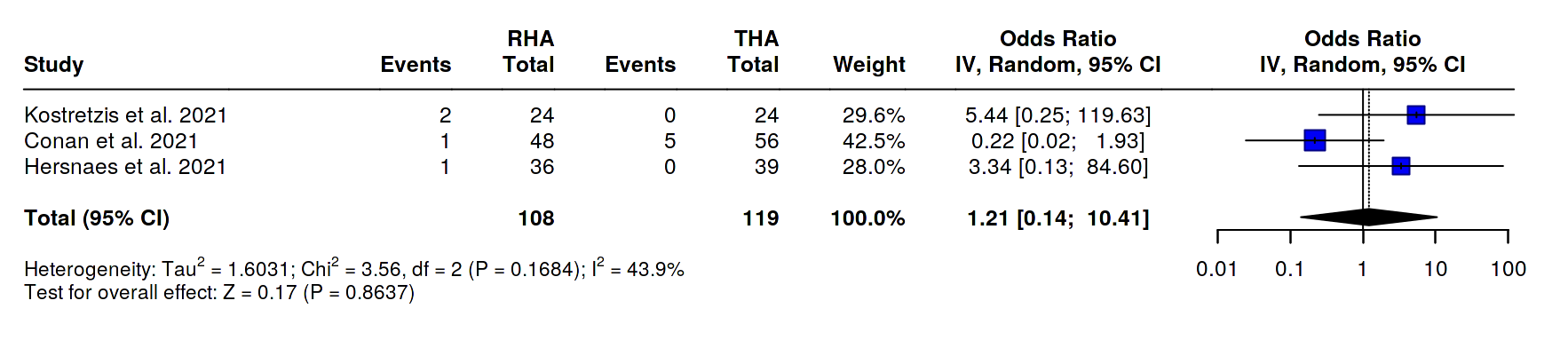


**Figure S2.24:** Forest plot of sensitivity analysis for revision rate excluding studies [19,23,26] predominantly utilizing smaller THA femoral head sizes (28-32mm).


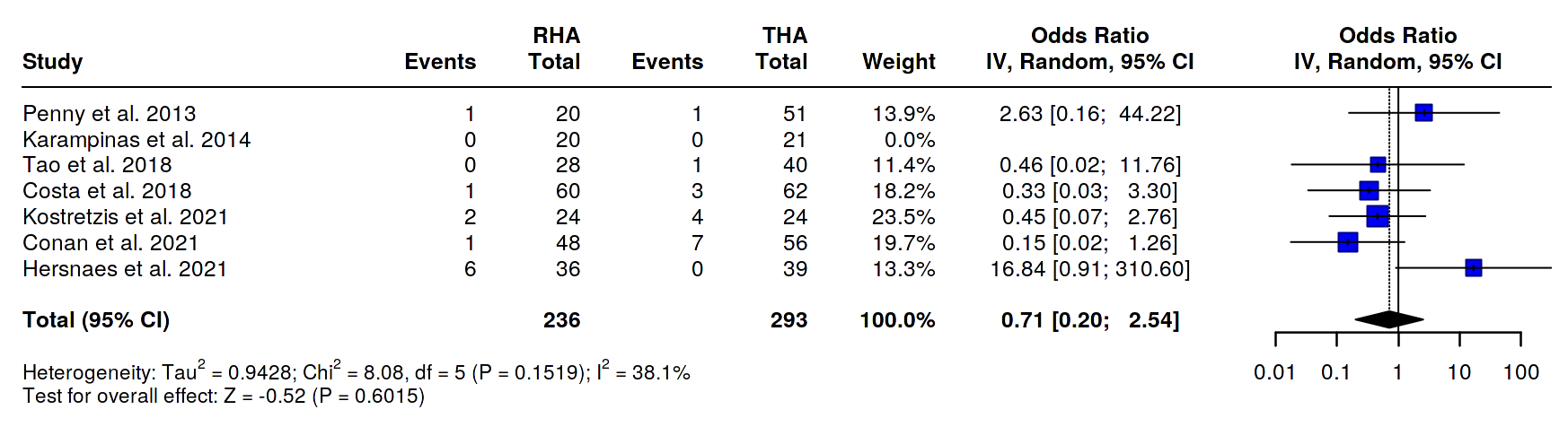


**Appendix S3: GRADE Assessment**

**a. Starting certainty of evidence**

For most pooled outcomes, the certainty of evidence initially started as **high,** because the greatest weight of evidence originated from randomised controlled trials. Outcomes were downgraded only where there were outcome-specific concerns regarding inconsistency, indirectness, imprecision, or other GRADE domains.

**b. Assumed and corresponding risks**
For dichotomous outcomes, the assumed risk represents the risk in the THA group. The corresponding risk with RHA was calculated from the pooled odds ratio using the formula:

Corresponding risk with RHA = (OR × assumed risk with THA) / [1 − assumed risk with THA + (OR × assumed risk with THA)]

Risks are presented per 1000 patients.

**c. Interpretation of statistical significance**
Statistical significance was not used as a separate GRADE domain. Instead, non-significant findings were considered under **imprecision,** based mainly on whether the 95% confidence interval crossed the line of no effect and whether it included clinically important benefit or harm.

**d. UCLA score**
Downgraded by two levels: one level for **inconsistency**, because heterogeneity was considerable, **I² = 78%,** and one level for **imprecision**, because the 95% CI crossed the line of no effect, **MD 0.44, 95% CI -0.02 to 0.90**.

**e. HHS score**
Downgraded by one level for **imprecision**, because the 95% CI crossed the line of no effect, **MD 1.54, 95% CI -0.06 to 3.14.** Although heterogeneity in the main analysis was moderate, **I² = 52%,** inconsistency was not downgraded because sensitivity analyses substantially reduced heterogeneity, suggesting that variability was partly explained by study design and population differences.

**f. WOMAC score**
Downgraded by one level for **imprecision**, because the 95% CI crossed the line of no effect, **MD -1.84, 95% CI -2.69 to 1.00.** Not downgraded for inconsistency because heterogeneity was low, **I² = 22%.**

**g. Cobalt ion levels**
Downgraded by two levels: one level for **inconsistency**, because heterogeneity was considerable, **I² = 86%,** and one level for **imprecision**, because the 95% CI crossed the line of no effect, **MD -0.48 μg/L, 95% CI -1.58 to 0.61.** Although sensitivity analyses reduced heterogeneity, only two studies remained, and the estimate was unstable.

**h. Chromium ion levels**
Downgraded by two levels: one level for **inconsistency**, because heterogeneity was substantial, **I² = 74%,** and one level for **imprecision**, because the 95% CI crossed the line of no effect, **MD 0.10 μg/L, 95% CI -0.76 to 0.96**. Although sensitivity analyses reduced heterogeneity, only two studies remained and the estimate changed, so inconsistency remained a concern.

**i. Total complications**
Downgraded by two levels: one level for **inconsistency**, because heterogeneity was substantial, **I² = 61%,** and one level for **imprecision**, because the 95% CI included both possible benefit and possible harm, **OR 0.66, 95% CI 0.25 to 1.73.**

**j. Fractures**
Downgraded by three levels to **very low certainty**: two levels for **very serious imprecision**, because the confidence interval was extremely wide and included both substantial benefit and substantial harm, **OR 0.50, 95% CI 0.05 to 4.70**, and one level for **inconsistency**, because heterogeneity was moderate/substantial, **I² = 57%.**

**k. Dislocations**
Downgraded by one level for **indirectness/applicability**, because the observed effect may be influenced by differences in THA femoral head size and bearing surface. No downgrade was applied for inconsistency because heterogeneity was absent, **I² = 0%.** No downgrade was applied for imprecision because the 95% CI did not cross the line of no effect, **OR 0.26, 95% CI 0.08 to 0.80.**

**l. ARMD**
Downgraded by two levels for **very serious imprecision**, because the confidence interval was very wide and included both possible benefit and possible harm**, OR 0.87, 95% CI 0.11 to 7.04**. Not downgraded for inconsistency because heterogeneity was not substantial, **I² = 38%**.

**m. Aseptic loosening**
Downgraded by two levels for **very serious imprecision**, because the confidence interval was very wide and included both no clear difference and a large possible increase in aseptic loosening with RHA, **OR 2.26, 95% CI 0.44 to 11.55**. Not downgraded for inconsistency because heterogeneity was not substantial, **I² = 38%.**

**n. Revision rates**
Downgraded by one level for **imprecision**, because the 95% CI crossed the line of no effect and included both possible benefit and possible harm, **OR 0.74, 95% CI 0.31 to 1.76**. No downgrade was applied for inconsistency because heterogeneity was low/moderate**, I² = 33%**.

**o. Survivorship**
Downgraded by two levels: one level for **indirectness**, because follow-up duration differed across studies and between treatment groups, and one level for **imprecision**, because no pooled comparative estimate with confidence intervals was calculated.

**p. Satisfaction and quality of life**
Downgraded by two levels: one level for **indirectness**, because different satisfaction and quality-of-life instruments were used across studies, and one level for **imprecision**, because findings were narratively synthesised, and no pooled comparative estimate was available.

**q. Publication bias**
Publication bias was considered qualitatively for each outcome. Formal assessment using funnel plots or statistical tests for asymmetry was not performed because most outcomes included fewer than 10 studies, making such methods unreliable. The review included searches of PubMed, EMBASE, and the Cochrane Library, which reduces but does not eliminate the possibility of publication bias. Publication bias was therefore not downgraded unless there was specific evidence suggesting missing or selectively reported studies. However, publication bias cannot be excluded, particularly for outcomes informed by few studies.

**r. Interpretation of certainty ratings**
**High certainty** indicates that further research is very unlikely to change confidence in the estimate of effect. **Moderate certainty** indicates that further research may have an important impact on confidence in the estimate. **Low certainty** indicates that further research is likely to have an important impact and may change the estimate. **Very low certainty** indicates that the true effect is very uncertain.

**Appendix S4: PRISMA Checklist**

| **Section and Topic** | **Item #** | **Checklist item** | **Location where item is reported** |
| --- | --- | --- | --- |
| **TITLE** | | |  |
| Title | 1 | Identify the report as a systematic review. | Page 1 |
| **ABSTRACT** | | |  |
| Abstract | 2 | See the PRISMA 2020 for Abstracts checklist. | Page 2 |
| **INTRODUCTION** | | |  |
| Rationale | 3 | Describe the rationale for the review in the context of existing knowledge. | Page 3 |
| Objectives | 4 | Provide an explicit statement of the objective(s) or question(s) the review addresses. | Page 3 |
| **METHODS** | | |  |
| Eligibility criteria | 5 | Specify the inclusion and exclusion criteria for the review and how studies were grouped for the syntheses. | Page 4 |
| Information sources | 6 | Specify all databases, registers, websites, organisations, reference lists and other sources searched or consulted to identify studies. Specify the date when each source was last searched or consulted. | Page 4 |
| Search strategy | 7 | Present the full search strategies for all databases, registers and websites, including any filters and limits used. | Page 4 |
| Selection process | 8 | Specify the methods used to decide whether a study met the inclusion criteria of the review, including how many reviewers screened each record and each report retrieved, whether they worked independently, and if applicable, details of automation tools used in the process. | Pages 4-5 |
| Data collection process | 9 | Specify the methods used to collect data from reports, including how many reviewers collected data from each report, whether they worked independently, any processes for obtaining or confirming data from study investigators, and if applicable, details of automation tools used in the process. | Pages 4-5 |
| Data items | 10a | List and define all outcomes for which data were sought. Specify whether all results that were compatible with each outcome domain in each study were sought (e.g. for all measures, time points, analyses), and if not, the methods used to decide which results to collect. | Pages 4, 5 |
|  | 10b | List and define all other variables for which data were sought (e.g. participant and intervention characteristics, funding sources). Describe any assumptions made about any missing or unclear information. | Pages 4-5 |
| Study risk of bias assessment | 11 | Specify the methods used to assess risk of bias in the included studies, including details of the tool(s) used, how many reviewers assessed each study and whether they worked independently, and if applicable, details of automation tools used in the process. | Page 5 |
| Effect measures | 12 | Specify for each outcome the effect measure(s) (e.g. risk ratio, mean difference) used in the synthesis or presentation of results. | Page 5 |
| Synthesis methods | 13a | Describe the processes used to decide which studies were eligible for each synthesis (e.g. tabulating the study intervention characteristics and comparing against the planned groups for each synthesis (item #5)). | Pages 4-5 |
|  | 13b | Describe any methods required to prepare the data for presentation or synthesis, such as handling of missing summary statistics, or data conversions. | Page 5 |
|  | 13c | Describe any methods used to tabulate or visually display results of individual studies and syntheses. | Page 5; Tables 5–8, pages 13–22; Figures 2–12, pages 11–19 |
|  | 13d | Describe any methods used to synthesize results and provide a rationale for the choice(s). If meta-analysis was performed, describe the model(s), method(s) to identify the presence and extent of statistical heterogeneity, and software package(s) used. | Page 5 |
|  | 13e | Describe any methods used to explore possible causes of heterogeneity among study results (e.g. subgroup analysis, meta-regression). | Page 5 |
|  | 13f | Describe any sensitivity analyses conducted to assess robustness of the synthesized results. | Page 5 |
| Reporting bias assessment | 14 | Describe any methods used to assess risk of bias due to missing results in a synthesis (arising from reporting biases). | N/A |
| Certainty assessment | 15 | Describe any methods used to assess certainty (or confidence) in the body of evidence for an outcome. | Pages 5-6 |
| **RESULTS** | | |  |
| Study selection | 16a | Describe the results of the search and selection process, from the number of records identified in the search to the number of studies included in the review, ideally using a flow diagram. | Page 6 |
|  | 16b | Cite studies that might appear to meet the inclusion criteria, but which were excluded, and explain why they were excluded. | Page 6 |
| Study characteristics | 17 | Cite each included study and present its characteristics. | Pages 7-8 |
| Risk of bias in studies | 18 | Present assessments of risk of bias for each included study. | Page 9 |
| Results of individual studies | 19 | For all outcomes, present, for each study: (a) summary statistics for each group (where appropriate) and (b) an effect estimate and its precision (e.g. confidence/credible interval), ideally using structured tables or plots. | Pages 11-22 |
| Results of syntheses | 20a | For each synthesis, briefly summarise the characteristics and risk of bias among contributing studies. | Pages 10-22 |
|  | 20b | Present results of all statistical syntheses conducted. If meta-analysis was done, present for each the summary estimate and its precision (e.g. confidence/credible interval) and measures of statistical heterogeneity. If comparing groups, describe the direction of the effect. | Pages 11-19, 23-26 |
|  | 20c | Present results of all investigations of possible causes of heterogeneity among study results. | Pages 22-26 |
|  | 20d | Present results of all sensitivity analyses conducted to assess the robustness of the synthesized results. | Pages 22-26 |
| Reporting biases | 21 | Present assessments of risk of bias due to missing results (arising from reporting biases) for each synthesis assessed. | N/A |
| Certainty of evidence | 22 | Present assessments of certainty (or confidence) in the body of evidence for each outcome assessed. | Pages 26 |
| **DISCUSSION** | | |  |
| Discussion | 23a | Provide a general interpretation of the results in the context of other evidence. | Pages 28-29 |
|  | 23b | Discuss any limitations of the evidence included in the review. | Pages 30-31 |
|  | 23c | Discuss any limitations of the review processes used. | Pages 30-31 |
|  | 23d | Discuss implications of the results for practice, policy, and future research. | Pages 28-31 |
| **OTHER INFORMATION** | | |  |
| Registration and protocol | 24a | Provide registration information for the review, including register name and registration number, or state that the review was not registered. | Page 4 |
|  | 24b | Indicate where the review protocol can be accessed, or state that a protocol was not prepared. | Page 4 |
|  | 24c | Describe and explain any amendments to information provided at registration or in the protocol. | N/A |
| Support | 25 | Describe sources of financial or non-financial support for the review, and the role of the funders or sponsors in the review. | Page 32 |
| Competing interests | 26 | Declare any competing interests of review authors. | Page 32 |
| Availability of data, code and other materials | 27 | Report which of the following are publicly available and where they can be found: template data collection forms; data extracted from included studies; data used for all analyses; analytic code; any other materials used in the review. | Page 32 |

*From:*  Page MJ, McKenzie JE, Bossuyt PM, Boutron I, Hoffmann TC, Mulrow CD, et al. The PRISMA 2020 statement: an updated guideline for reporting systematic reviews. BMJ 2021;372:n71. doi: 10.1136/bmj.n71. This work is licensed under CC BY 4.0. To view a copy of this license, visit <https://creativecommons.org/licenses/by/4.0/>
